# Deep learning–based CT lesion analysis and radiological burden stratification in clinically diagnosed ventilator-associated pneumonia

**DOI:** 10.3389/fmed.2026.1966805

**Published:** 2026-10-23

**Authors:** Fuwen Zheng, Andee Dzulkarnaen Zakaria

**Affiliations:** School of Medical Sciences, Universiti Sains Malaysia, Health Campus, Kubang Kerian, Kelantan, Malaysia

**Keywords:** chest CT, deep learning, multi-task learning, severity grading, ventilator-associated pneumonia

## Abstract

Ventilator associated pneumonia (VAP) is a major complication in the intensive care unit (ICU) and requires objective computed tomography (CT) evaluation. Therefore, this article conducted a retrospective research analysis and developed a multimodal deep learning framework. Firstly, this retrospective study included 512 patients clinically diagnosed with VAP, and then utilized a developed multimodal deep learning framework for voxel-level lesion segmentation, multi-label radiological discovery classification, and radiological burden grading. This framework integrates the anatomical priors of TotalSegmenter, a three-dimensional (3D) Swin UNETR multitasking network, and cross attention fusion of CT features and ventilation parameters. Ultimately, the study showed that the Dice score for internal validation was 0.847, the macroscopic mean area (AUC) under the subject's working characteristic curve was 0.919, and the weighted kappa was 0.826. External validation showed a Dice score of 0.821, with no significant difference between scanner manufacturers. After adjusting for clinical covariates, including acute physiology and chronic health assessment II (APACHE II) scores, there was still a significant correlation between radiation load level and 28 day mortality (hazard ratio [HR] = 2.34, *P* < 0.001), supporting its potential prognostic relevance.

## 1 Introduction

Ventilator-associated pneumonia (VAP) refers to a new parenchymal lung infection that occurs in patients who have undergone endotracheal intubation or tracheotomy 48 h after receiving mechanical ventilation. Its incidence rate in mechanically ventilated ICU patients is as high as 9%–27%, with an attributable mortality rate between 20% and 50%. It prolongs the average ICU stay by 6–13 days and increases the treatment cost per case by approximately US$40,000, making it one of the most challenging nosocomial infection problems in the global critical care medicine field (1, 2). The clinical diagnosis of VAP has long been constrained by three major bottlenecks: the sensitivity and specificity of the Clinical Pulmonary Infection Score (CPIS) are both below 70%; pathogen culture has a diagnostic lag of 48–72 h; and bedside chest X-rays are limited by resolution and patient positioning, making it difficult to accurately assess the extent of lesions. Chest CT has become the gold standard for VAP imaging assessment due to its high resolution of dorsal consolidation, barotrauma changes, and necrotic cavitation in gravity-dependent areas (3, 4). However, problems such as strong subjectivity in image interpretation, blurred lesion boundaries, and lack of quantitative standards for severity make the construction of an objective and reproducible intelligent auxiliary diagnostic tool an urgent clinical need with far-reaching application value.

Recent advances in artificial intelligence have created new opportunities for quantitative analysis of thoracic CT by enabling automated lesion localization, feature recognition, and disease-burden assessment. Nevertheless, VAP remains a particularly challenging application scenario because its CT manifestations are often heterogeneous, spatially diffuse, and easily confounded by ICU-related devices and concurrent pulmonary abnormalities. In addition, radiological findings alone may not fully reflect the physiological consequences of pulmonary infection in mechanically ventilated patients. These characteristics indicate that a clinically meaningful VAP-oriented framework should not rely solely on image-level pattern recognition, but should integrate anatomical context, lesion-level quantitative analysis, multi-task representation learning, and complementary respiratory physiological information within a unified model.

To address the aforementioned research gaps, this paper develops a deep-learning framework for quantitative CT lesion analysis and radiological burden stratification in patients with clinically diagnosed VAP. The main research content covers four aspects. First, a CT image database containing 512 confirmed VAP cases is constructed based on a retrospective cohort. The present cohort contains only patients who met the predefined clinical and etiological criteria for VAP; therefore, the study is not designed to distinguish VAP from non-VAP pulmonary conditions. Double-blind annotation and a third-party arbitration mechanism are used to complete voxel-level annotation of five radiological findings: ground-glass opacity, consolidation, centrilobular nodules, cavitation, and pleural effusion. Second, a two-stage cascaded network architecture is designed. The first stage extracts anatomical priors of lung lobes based on TotalSegmentator and removes ICU instrument artifacts. The second stage uses 3D Swin UNETR as the backbone and integrates an nnU-Net self-configuration strategy to construct a multi-task system for lesion segmentation, radiological-sign classification, and radiological burden stratification. Third, a multimodal fusion mechanism of image and ventilation parameters is proposed, deeply coupling clinical parameters such as PEEP and oxygenation index with visual features through Cross-Attention. Fourth, a three-level ordered classification system of severity is established based on lesion volume proportion and the number of lung lobes involved, and its clinical prognostic value is verified by its correlation with 28-day mortality. The principal methodological contribution of this study lies not in proposing each individual architectural component as a new algorithm, but in their task-oriented integration for quantitative VAP CT analysis. Specifically, the framework combines anatomical-prior-guided 3D image analysis, multi-task learning, and cross-attention-based fusion of CT features with mechanical ventilation parameters to jointly support lesion segmentation, radiological-finding classification, and radiological burden stratification.

## 2 Related work

In recent years, deep learning for medical images has undergone a paradigm shift from convolutional architecture to Transformer architecture. U-Net, proposed by Ronneberger et al. (5) in 2015, laid the foundation for the encoder-decoder framework of medical image segmentation. nnU-Net, proposed by Isensee et al. (6) in 2021, achieved robust performance across tasks through a self-configuration mechanism, becoming a strong baseline in the field of medical segmentation. With the rise of Vision Transformer, Hatamizadeh et al. (7) proposed UNETR (2022), introducing the hierarchical window attention mechanism of Swin Transformer into 3D medical image segmentation, achieving significant improvements on the benchmark. Medical basic models represented by SAM-Med3D (Segment Anything Model for 3D Medical Images) (8) further promoted zero-shot and few-shot segmentation capabilities. In the field of intelligent analysis of pneumonia images, the COVID-19 (Coronavirus Disease 2019) pandemic has spurred a large number of research efforts: Morozov et al. (9) published MosMedData, which constructed a CT dataset with severity grading; Wang et al. (10) proposed COVID-Net, which realized end-to-end pneumonia screening; Shan et al. (11) developed VB-Net (Volume-Based Network), which realized automated CT severity scoring of pneumonia lesions. These works have accumulated an important technical foundation for the intelligent analysis of pulmonary infectious diseases.

Despite significant progress in intelligent analysis of pneumonia CT scans, research on VAP, a specific clinical scenario, remains largely unexplored. Existing public datasets (MosMedData, COVID-19-CT-Seg, RADLogics, etc.,) (12–14) all focus on community-acquired pneumonia or COVID-19. However, VAP, as an in-hospital-acquired infection, exhibits significant scenario-specific imaging characteristics: prolonged bed rest in mechanically ventilated patients leads to prominent dorsal consolidation in gravity-dependent areas; diffuse ground-glass opacities in the context of ARDS (Acute Respiratory Distress Syndrome) are highly mixed with actual infection foci; ICU instruments such as endotracheal tubes, gastric tubes, and ECG leads create artifacts in CT images; and barotrauma-related mediastinal emphysema, pneumothorax, and atelectasis make lesion boundary determination more difficult (15, 16). Existing studies have shown that directly transferring pneumonia segmentation models pre-trained on COVID-19 data to the VAP scenario generally results in a decrease in the Dice coefficient, indicating limited cross-scenario generalization ability. Furthermore, existing pneumonia severity scoring systems are mostly based on a single imaging dimension, lacking multimodal fusion with ventilation parameters, and few studies have verified the correlation between the model's output grading results and key clinical outcomes such as ICU 28-day mortality rate, which restricts the transformation of intelligent diagnostic tools from technically feasible to clinically usable.

Recent advances in medical artificial intelligence have further shifted the field from isolated image analysis toward multimodal representation learning, adaptive feature fusion, and collaborative multi-task modeling. Gao et al. (17) demonstrated that integrating CT-derived imaging representations with complementary clinical information can support simultaneous disease screening, staging, and progression-risk stratification, highlighting the potential value of imaging–clinical multimodal fusion. Huang et al. (18) introduced text–image co-alignment to strengthen semantic guidance for weakly supervised medical image segmentation, while Hong et al. (19) showed that adaptive weighted fusion can effectively integrate complementary information from sparse multi-view X-ray observations. At the task level, Li et al. (20) demonstrated that segmentation can serve as a structural prior for another clinically relevant imaging task, supporting the benefits of collaborative multi-task learning. Deep-learning-based denoising and super-resolution studies by Yu et al. (21) and Luan et al. (22) further illustrate the importance of robust feature extraction and image-quality enhancement for biomedical imaging under imperfect acquisition conditions. From a pulmonary pathophysiological perspective, Yuan et al. (23) highlighted the roles of excessive inflammatory activation, oxidative stress, and mitochondrial dysfunction in severe pneumonia-associated lung injury. Collectively, these developments support the integration of adaptive multimodal fusion, task-level collaboration, and robust image representation; however, their systematic application to quantitative CT lesion analysis and outcome-oriented stratification in VAP remains insufficiently explored.

## 3 Methods

### 3.1 Overall framework

As shown in Figure 1, the proposed framework follows a two-stage cascaded architecture. The input consists of a three-dimensional chest CT volume and a five-dimensional clinical vector containing PEEP, PaO₂/FiO₂ ratio, ventilation duration, tidal volume, and FiO₂. CT volumes are preprocessed by HU clipping, isotropic resampling, and data augmentation. In the first stage, pretrained TotalSegmentator is used to extract five lung-lobe masks and an airway mask, which are combined with the artifact-suppressed CT volume to construct the seven-channel input for the second-stage network.

**Figure 1:**
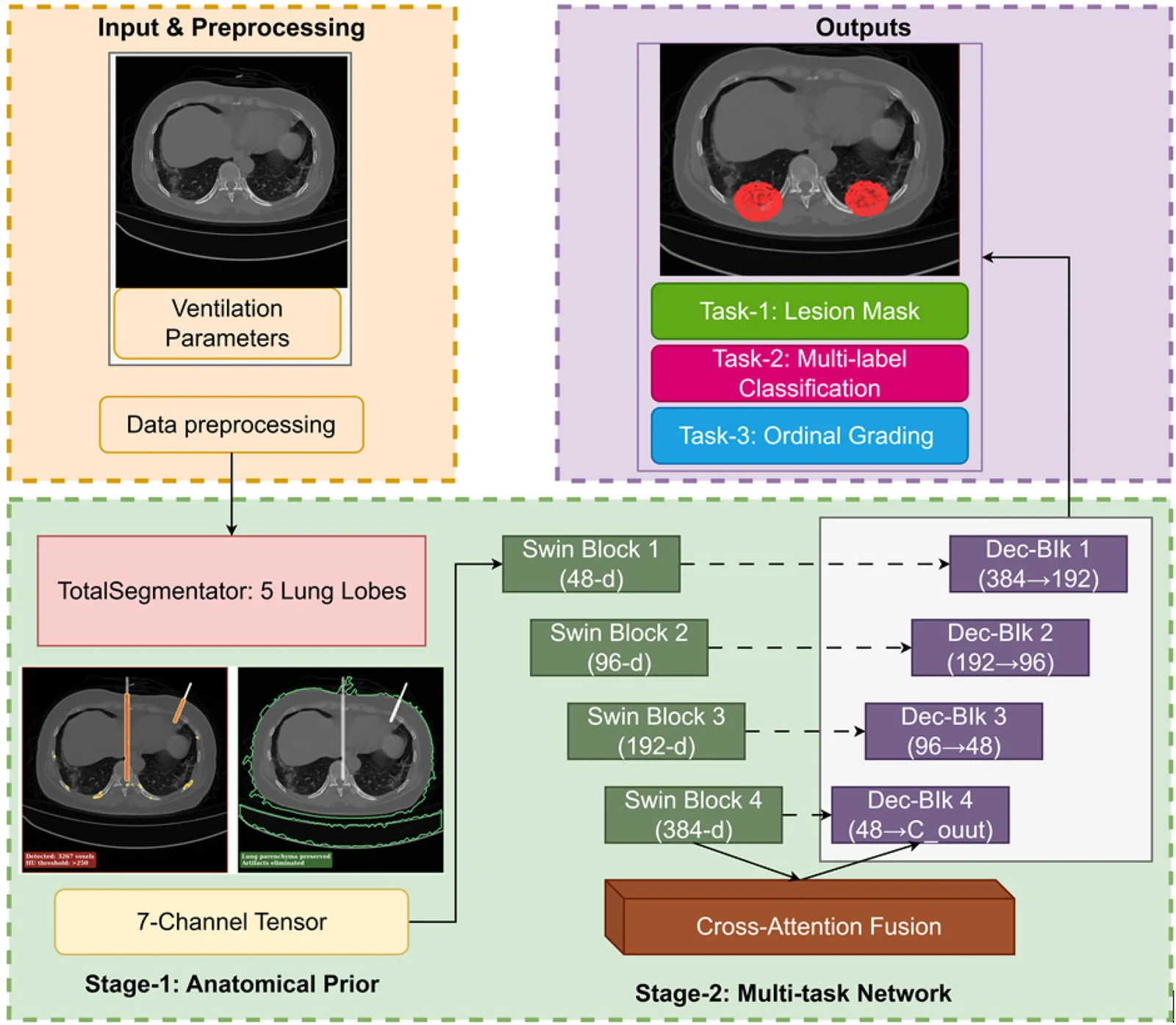
Overall framework for CT lesion analysis and radiological burden stratification in clinically diagnosed VAP.

As shown in Figure 1, the proposed CT lesion analysis and radiological burden stratification framework adopts an end-to-end cascaded architecture an end-to-end cascaded architecture: the data input layer simultaneously receives the three-dimensional chest CT volume (512 × 512 × N, slice thickness 1.0–1.25 mm, lung window W1500/L-600) and a five-dimensional ventilation parameter clinical vector consisting of PEEP, oxygenation index P/F, ventilation duration, tidal volume, and FiO₂, realizing heterogeneous fusion of imaging and physiological information. The preprocessing stage performs HU (Hounsfield Unit) value truncation [−1000, 400], isotropic resampling to 1 × 1 × 1 mm^3^, and multi-strategy data enhancement including elastic deformation and MixUp. The first stage (anatomical prior extraction) uses a pre-trained TotalSegmentator to accurately segment the five lobes of the lung (RUL/RML/RLL/LUL/LLL) (Right Upper Lobe/Right Middle Lobe/Right Lower Lobe/Left Upper Lobe/Left Lower Lobe), and uses HU thresholding combined with morphological filtering to remove artifacts of ICU instruments such as endotracheal tubes, and finally constructs a multi-channel input tensor with dimensions of 7 × 96 × 96 × 96. The second stage (multi-task network) uses a four-level hierarchical 3D Swin Transformer as the encoder backbone (feature dimensions 48→96→192→384-d). At the bottleneck, Cross-Attention is used to deeply couple the 128-dimensional clinical features encoded by the Clinical MLP (Multi-Layer Perceptron) with visual features through a Q/K/V triple mechanism. The decoder upsamples step by step through skip connections and branches into three task heads. The output layer produces a voxel-level lesion segmentation mask, multi-label classification probabilities of five types of signs, and an ordered three-level severity rating (mild/moderate/severe) based on the CORAL framework.

### 3.2 Data acquisition and preprocessing

#### 3.2.1 Data sources and inclusion/exclusion criteria

This study employed a single-center retrospective cohort design. All chest CT imaging data were obtained from inpatients receiving invasive mechanical ventilation in the Intensive Care Unit (ICU) of a collaborating tertiary hospital between January 2020 and June 2024. Initial screening was conducted through a combined search of the electronic medical record system and the radiology information system, using “mechanical ventilation,” “VAP,” and “hospital-acquired pneumonia” as keywords, initially identifying 874 candidate cases. Subsequent manual review based on strict inclusion and exclusion criteria resulted in the final enrollment of 512 cases.

The inclusion criteria (24, 25) include the following three dimensions: (1) Ventilation time criteria: the duration of invasive mechanical ventilation after endotracheal intubation or tracheotomy is ≥48 h, or new signs of pneumonia are found within 48 h of weaning/extubation; (2) Clinical diagnostic criteria: new or progressive pulmonary infiltrates accompanied by fever (body temperature >38.0℃) or abnormal white blood cell count (>12 × 10⁹/L or <4 × 10⁹/L) and purulent airway secretions; (3) Etiological evidence: positive quantitative culture of lower respiratory tract secretions and colony count reaching the diagnostic threshold. Exclusion criteria include: (1) patients with severe motion artifacts or metal implant artifacts on CT images that make lung parenchyma interpretation difficult; (2) non-thin slice scans with a slice thickness >5 mm or uneven slice spacing; (3) patients with structural lung diseases such as primary or metastatic lung tumors, tuberculosis, or pneumoconiosis that may interfere with lesion interpretation; (4) patients under 18 years of age or pregnant patients; and (5) patients diagnosed with pneumonia before mechanical ventilation, to ensure the etiological specificity of VAP.

The external validation cohort was also retrospectively collected from an independent collaborating tertiary hospital using the same predefined inclusion and exclusion criteria. No prospective patient recruitment or data acquisition was performed for the external cohort.

#### 3.2.2 Standardization of CT scanning parameters

This study implemented strict standardization control over the scanning and reconstruction parameters of the enrolled CT images. All CT examinations were performed using three mainstream in-house CT scanners: GE Revolution CT (64-slice, 128-layer), Siemens SOMATOM Definition Flash (dual-source, 128-slice), and Philips Brilliance iCT (256-slice). The patient was positioned supine with a deep inspiration and breath-hold at the end, or during mechanical ventilation with paused acquisition of the last phase. The scanning range extended from the thoracic inlet to the lower edge of the diaphragm. The following parameters for the scan: tube voltage 120 kVp, current in the range of 100–300 mAs using automatic adjustment, pitch 0.984–1.375, gantry rotation time 0.5 s, matrix 512 × 512, and field of view 350 mm.

In the realm of medicine, reconstructions of images, especially pertaining to the lungs, require striking a balance between capturing both high-frequency detail and low-frequency density information of lung lesions. To achieve this duality, a dual-kernel reconstruction methodology consisting of a standard B30f/B40f convolutional kernel for soft tissue windows and a high-resolution B70f/B80f sharp convolutional kernel is employed for lung parenchyma analysis. All slices uniformly at 1.0–1.25 mm slice thickness with equal interslice spacing between contiguous slices are reconstructed to allow for seamless contiguous reconstructions. For lesion evaluation and annotation, lung window settings of W = 1500HU and L = −600HU are used. Then, mediastinal windows (W = 400HU and L = 40HU) are employed as reference points to help differentiate between pleural and pericardial visceral and parietal effusions. Patient identification information is stripped from images using anonymized code names for renaming and only research-related metadata regarding scan parameter files is maintained during this process.

At the development institution, CT examinations were acquired using GE Revolution CT, Siemens SOMATOM Definition Flash, and Philips Brilliance iCT scanners. The standardized acquisition protocol used a tube voltage of 120 kVp, automatic tube-current modulation within 100–300 mAs, a pitch of 0.984–1.375, a gantry rotation time of 0.5 s, and a 512 × 512 reconstruction matrix. Images were reconstructed at a slice thickness of 1.0–1.25 mm using both standard and high-resolution lung kernels. At the external institution, the 77 CT examinations were acquired on independent scanners from GE (*n* = 28), Siemens (*n* = 26), and Philips (*n* = 23). The same eligibility criteria and image-standardization pipeline, including HU normalization and isotropic resampling, were applied before external testing.

#### 3.2.3 Preprocessing flow

The complete preprocessing flow consists of five sequential steps: HU value truncation (26, 27), intensity normalization, isotropic resampling, lung field ROI (Region of Interest) extraction, and data augmentation.

The original CT values have a wide dynamic range (−3000 to +3000 HU), but VAP lesions mainly present as ground-glass opacities (−700 to −300 HU), consolidation (−100 to +50 HU), and cavitation (−1000 to −500 HU) (28, 29).

The preprocessing pipeline was performed in the following order: high-density device artifact identification and suppression on the original, unclipped CT volume, followed by HU value truncation, intensity normalization, isotropic resampling, lung-field ROI extraction, and data augmentation. Importantly, artifact detection was completed before HU clipping to preserve the original high-density information required for device identification.

Therefore, the HU values are truncated to the range of [−1000, 400] to remove irrelevant high-density signals such as bone and metal instruments. The formula is expressed as the following Equation 1:

$$I_{clip}(x,y,z)=\min(\max(I_{raw}(x,y,z)\text{-}1000),400)$$ (1)

Z-score normalization is used to map the truncated voxel values to a zero-mean unit variance distribution, so that the gray-scale differences between different scanners can be eliminated:

$$I_{norm}(x,y,z)=\frac{I_{clip}(x,y,z)-\mu_{I}}{\sigma_{I}+\in }$$ (2)

In the Equation 2, $\mu_{I}$ and $\sigma_{I}$ are the mean and standard deviation of voxels in the lung fields, respectively, and $\epsilon =10^{\text{-}8}$ is used to prevent division by zero anomalies.

Given the non-uniformity of slice thickness (1.0/1.25 mm) and in-plane pixel spacing (0.6–0.8 mm) across different devices, trilinear interpolation was used to resample all CT volumes to an isotropic resolution of 1 × 1×1 mm^3^ to ensure consistency of the receptive field of the 3D convolution kernel across the three spatial dimensions.

Lung parenchyma was separated based on threshold segmentation (−500 HU) combined with 3D connected component analysis and morphological closing operations (5 mm radius spherical structuring element), eliminating non-interest regions such as the chest wall, mediastinum, and abdominal cavity. During the training phase, an online augmentation strategy was employed to enhance the model's generalization ability. This includes: random 3D rotation (±15°), elastic deformation (Gaussian kernel *σ* = 8, amplitude *α* = 500), Gamma correction (*γ*∈[0.7, 1.5]), random flipping (sagittal plane), Gaussian noise injection (*σ* = 0.01), and a MixUp sample mixing strategy:

$$\tilde{x}=\lambda x_{i}+(1\text{-}\lambda )x_{j},\tilde{y}=\lambda y_{i}+(1\text{-}\lambda )y_{j},\lambda ∼\mathrm{Beta}(0.4,0.4)$$ (3)

In the Equation 3, $\left(x_{i},y_{i}\right)$ and $\left(x_{j},y_{j}\right)$ are two randomly selected training sample pairs.

#### 3.2.4 Annotation standards

This study employed a three-tiered annotation procedure consisting of independent dual-reader annotation, third-party arbitration, and senior review. The annotation team consisted of five radiologists with thoracic imaging specialties. Two associate chief physicians (with 12 and 15 years of clinical experience respectively) served as independent primary annotators, two chief physicians (with over 20 years of experience) served as third-party arbitrators, and one physician with a background in both ICU and radiology served as the final reviewer.

The annotations covered five typical CT features related to VAP: (1) Ground-Glass Opacity (GGO) – mild increase in lung density that does not obscure the course of pulmonary vessels and bronchi; (2) Consolidation – increased lung density to the point of obscuring vascular shadows, often accompanied by air bronchograms; (3) Centrilobular Nodule – tree-in-bud appearance commonly seen around bronchioles; (4) Cavity – a radiolucent area formed by necrosis within consolidation; (5) Pleural Effusion. The two primary radiologists were aware that all included patients had a clinical diagnosis of VAP, but they performed the CT annotations independently and were blinded to each other’s annotations. The availability of microbiological results, laboratory findings, previous imaging, ventilation-related information, and patient outcomes was controlled during annotation to prevent these data from influencing lesion delineation. Interobserver agreement was assessed for the voxel-level annotations before arbitration, yielding an overall Cohen’s κ of 0.81 (95% CI: 0.78–0.84).

$$\kappa =\frac{\;p_{o}-p_{e}}{1-p_{e}}$$ (4)

In the Equation 4, $p_{o}$ represents the proportion of observations agreed upon by the two annotators, and $p_{e}$ represents the expected proportion of random agreement.

Importantly, the radiologist annotation procedure in this study was restricted to lesion-level delineation and sign-level identification of ground-glass opacity, consolidation, centrilobular nodules, cavitation, and pleural effusion. The radiologists did not independently assign case-level mild, moderate, or severe VAP categories. Accordingly, the three-level target used for the grading task should not be interpreted as an independent radiologist-derived clinical severity reference standard. Instead, it represents an algorithmically defined radiological burden category derived from quantitative CT characteristics obtained from the adjudicated lesion annotations.

To visually demonstrate the data preprocessing and annotation workflow of this study, Figure 2 presents the complete processing chain of original CT, HU truncation, lung field ROI extraction, isotropic resampling, and voxel-level annotation of the five sign categories.

**Figure 2:**
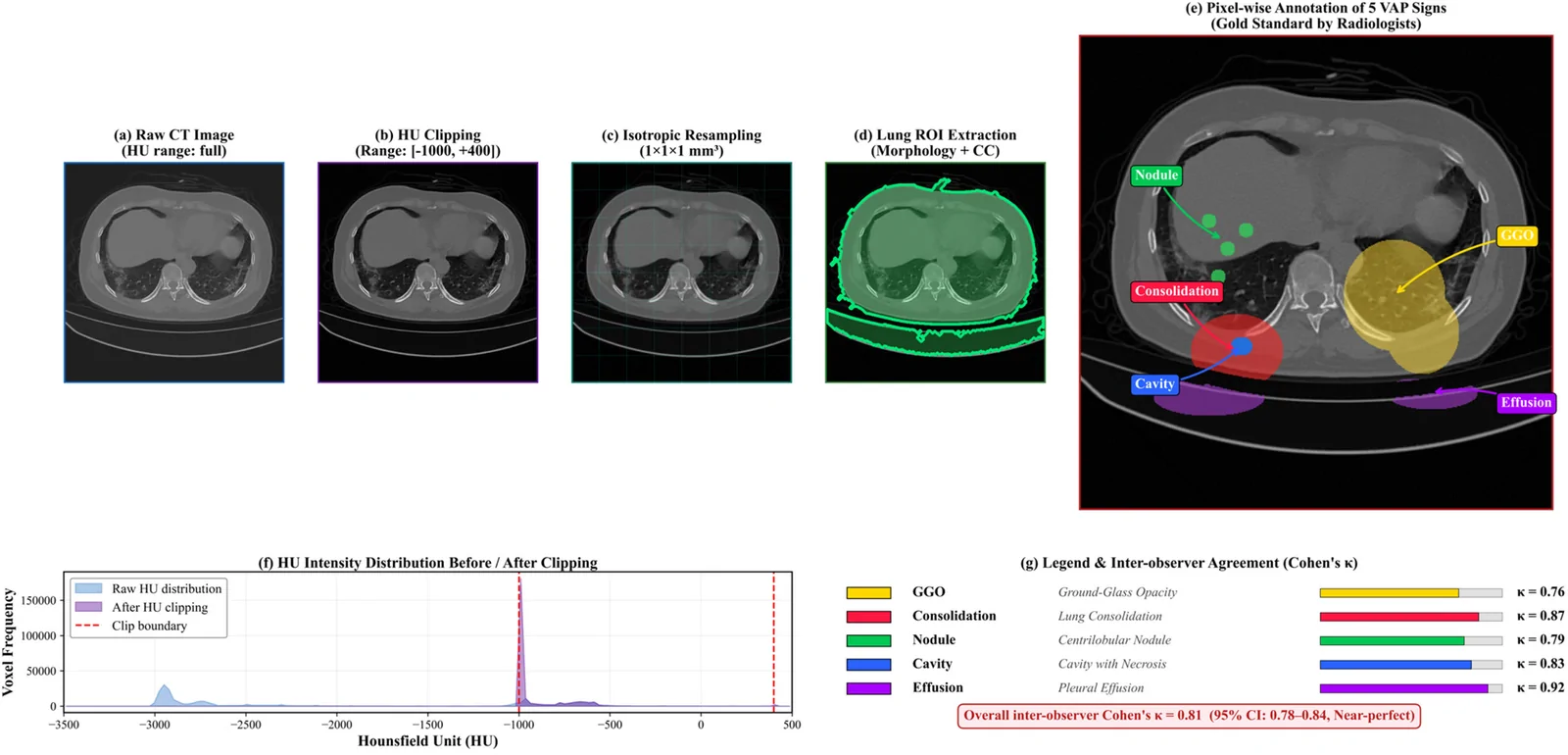
Examples of data preprocessing and annotation. **(a)** Raw CT image (HU range: full); **(b)** HU clipping (range: [-1000, +400]); **(c)** isotropic resampling (1×1×1 mm_3_); **(d)** lung ROI extraction (morphology +CC); **(e)** pixel-wise annotation of 5 VAP signs (gold standard by radiologists); **(f)** HU intensity distribution before / after clipping; **(g)** legend & inter-observer agreement (Cohen's *k*).

Figure 2a shows the wide dynamic range (HU range up to [−3000, +3000]) of the original CT, containing a large amount of irrelevant high-density signals. Figure 2b shows the dynamic range compressed to [−1000, +400] after HU truncation, effectively suppressing bone and metal instrument artifacts. Figure 2c shows the spatial consistency of the 3D convolutional receptive field by trilinear interpolation resampling to an isotropic 1 × 1 × 1 mm^3^ resolution. Figure 2d shows the accurate extraction of bilateral lung parenchymal ROIs (green outlines and translucent covering layers) through threshold segmentation combined with morphological closure operations and connected component analysis. Figure 2e presents the voxel-level gold standard of five typical radiological findings annotated by radiologists, including ground-glass opacity (yellow), consolidation (red), centrilobular nodules (green), cavitation (blue), and pleural effusion (purple). Figure 2f shows the quantitative comparison of voxel intensity distribution before and after truncation using the HU histogram. Figure 2g shows the inter-observer consistency of the double-blind labeled data. Overall, Cohen's *κ* = 0.81 (95% CI: 0.78–0.84). Among the data, pleural effusion showed the highest consistency (*κ* = 0.92), while GGO showed the lowest consistency due to its ambiguous boundaries (*κ* = 0.76). This is consistent with the objective laws of clinical sign interpretation and provides high-quality gold standard data for subsequent model training.

### 3.3 First stage: anatomical structure segmentation and prior extraction

#### 3.3.1 Transfer application of TotalSegmentator

To address the core challenges of blurred boundaries between lesions and background tissues in chest CT scans of VAP patients, severe interference from ICU instrument artifacts, and significant differences in involvement of different lung lobes, this paper designs an independent first-stage processing module before entering the main lesion segmentation network. Its core objective is to extract anatomical structure priors with clinical semantics, eliminate instrument artifacts in non-pulmonary parenchymal regions, and embed these structured priors into the input space of the second-stage network in a channel splicing manner.

TotalSegmentator is an open-source general medical image segmentation model trained on 1,204 multi-center whole-body CT data using the nnU-Net framework by Wasserthal et al. (30). It can automatically segment up to 117 anatomical structures in a single forward inference and has shown superior cross-dataset generalization ability in multiple anatomical regions such as the lungs, heart, and abdomen. This study loads the pretrained TotalSegmentator v2.0 weights and performs inference with all model parameters frozen. For the downstream VAP analysis, only six anatomical prior masks are retained: the right upper lobe (RUL), right middle lobe (RML), right lower lobe (RLL), left upper lobe (LUL), left lower lobe (LLL), and an airway mask derived from the trachea and main bronchi. The pulmonary artery segmentation is not used as an input channel or downstream feature in the proposed framework, formally defined as:

$$\{M_{\mathrm{RUL}},M_{\mathrm{RML}},M_{\mathrm{RLL}},M_{\mathrm{LUL}},M_{\mathrm{LLL}},M_{airway}\}=f_{\mathrm{TS}}(I_{CT};\theta^{*})$$ (5)

In the Equation 5, $I_{CT}\in R^{H\;\times W\;\times D}$ is the preprocessed CT volume; $f_{TS}$ is the TotalSegmentator network; and $\theta^{*}$ is the frozen pre-trained weights.

#### 3.3.2 ICU instrument artifact removal mechanism

CT images of mechanically ventilated patients inevitably contain high-density medical devices such as endotracheal tubes, gastric tubes, ECG leads, pulse oximeters, and central venous catheters. These appear on CT as high-HU (>600 HU) linear or dotted structures similar to bone (31, 32). Without processing, these artifacts can easily be misidentified by deep learning models as blood vessels, consolidation, or even calcifications, severely interfering with the reliability of lesion identification. Therefore, this study designed a three-level joint filtering strategy of “HU threshold—geometric morphology—topological connectivity” to achieve automatic detection and suppression of instrument artifacts.

Level 1: HU Threshold Screening. This screening was applied to the original CT volume before intensity clipping; therefore, the high-HU information required for artifact detection was fully preserved. After the final artifact mask was obtained, the identified voxels were replaced with −950 HU, and the artifact-suppressed volume was subsequently clipped to [−1000, 400] HU and normalized for network input. Based on the metallic or polymeric material characteristics of medical devices, their CT values are generally significantly higher than those of lung parenchyma (−1000 to −400 HU) and even soft tissue (−100 to +100 HU). Therefore, a threshold $T_{HU}=600$ HU was set as the initial screening condition for candidate artifact voxels:

$$M_{cand}(x,y,z)=1[I_{CT}(x,y,z)\;\mathrm{gt}T_{HU}]⊙(1-M_{lung}(x,y,z))$$ (6)

In the Equation 6, $M_{\mathrm{lung}}=\cup_{i=1}^{5}M_{\mathrm{lob}e_{i}}$ is the lung field mask for the combined five lobes of the lung; ⊙ represents the voxel-by-voxel product; and 1[⋅] is the indicator function. This operation removes high-density regions inside the lung field, retaining only high-density candidate voxels outside the lung field and in the thoracic cavity crossing area.

Second level: Geometric morphology discrimination. Real medical devices have significant elongated tubular or linear geometric features, while lesions are usually spherical or lumpy. In this study, three-dimensional skeletonization was used to extract the center line of the candidate region, and then its elongation index was calculated:

$$\mathrm{ER}(C_{k})=\frac{\lambda_{\max}(C_{k})}{\lambda_{\min}(C_{k})+\varepsilon }$$ (7)

In the Equation 7, $\lambda_{\max}$ and $\lambda_{\min}$ are the maximum and minimum eigenvalues of the covariance matrix of the voxel coordinates of the connected domain $C_{k}$, respectively, and $\varepsilon =10^{\text{-}6}$ is the numerical stability term. When $\mathrm{ER}(C_{k})\;\mathrm{gt}15$, it is judged as a tubular device; when $\mathrm{ER}(C_{k})\;\mathrm{gt}[5,15]$ and located within the tracheal anatomical path (based on the $M_{\mathrm{AW}}$ expansion zone), it is judged as tracheal intubation.

Level 3: Topological Connectivity and Spatial Consistency Constraints. Real-world instruments typically penetrate the body surface and extend into the body, exhibiting a topological characteristic of a single connected component connected to the body surface boundary. This study performed 3D connected component analysis on candidate regions selected through the first two levels, retaining only connected components connected to image boundaries or continuously distributed along the tracheal anatomical path to obtain the final instrument artifact mask $M_{\mathrm{dev}}$. During the network input construction stage, the voxel corresponding to $M_{\mathrm{dev}}$ was set to the lung parenchyma background HU value (−950 HU) to achieve soft suppression and avoid sharp boundary artifacts introduced by hard masks.

#### 3.3.3 Embedding of anatomical priors

To effectively transfer the anatomical information extracted in the first stage to the lesion recognition network in the second stage, this study abandons the traditional post-processing prior application method and instead adopts an early fusion channel splicing strategy, so that the anatomical prior can participate in feature learning in every convolutional layer of the network, thereby guiding the model's attention to focus on clinically relevant areas. Specifically, the six retained anatomical prior masks—five lung-lobe masks and one airway mask—are concatenated with the artifact-suppressed CT volume along the channel dimension, yielding the final seven-channel input tensor (1 CT channel + 5 lung-lobe channels + 1 airway channel). No pulmonary artery channel is included in the second-stage network.

$$X_{\mathrm{in}}=\mathrm{Conca}t_{c}[{\hat{I}}_{CT},M_{\mathrm{RUL}},M_{\mathrm{RML}},M_{\mathrm{RLL}},M_{\mathrm{LUL}},M_{\mathrm{LLL}},M_{\mathrm{AW}}]$$ (8)

In the Equation 8, ${\hat{I}}_{CT}$ is the normalized CT volume after instrument artifact suppression, and $\mathrm{Conca}t_{c}$ represents the tensor splicing operation along the channel dimension. The reasons for choosing this 7-channel design instead of merging the lung lobe masks into a single-channel multi-label map are: (1) each lung lobe, as an independent channel, can retain its spatial specific information, enabling the network to learn the differences in VAP involvement patterns among different lung lobes; (2) one-hot representation of independent channels avoids the ordinal bias problem of multi-label encoding; (3) airway channels, as independent inputs, facilitate the network's capture of special signs such as tree-bud signs around the trachea and bronchi.

### 3.4 Second stage: multi-task lesion segmentation–radiological sign classification–burden stratification network

After completing the anatomical prior extraction, the second stage undertakes the core tasks of fine identification and severity quantification of VAP lesions (33, 34). To simultaneously achieve the three complementary goals of voxel-level segmentation, sign-level multi-label recognition, and case-level severity grading, this study designed an end-to-end multi-task learning network based on a 3D Swin Transformer backbone and incorporating multimodal clinical parameters. Figure 3 fully presents the detailed architecture of this network and the information flow topology between its modules.

**Figure 3:**
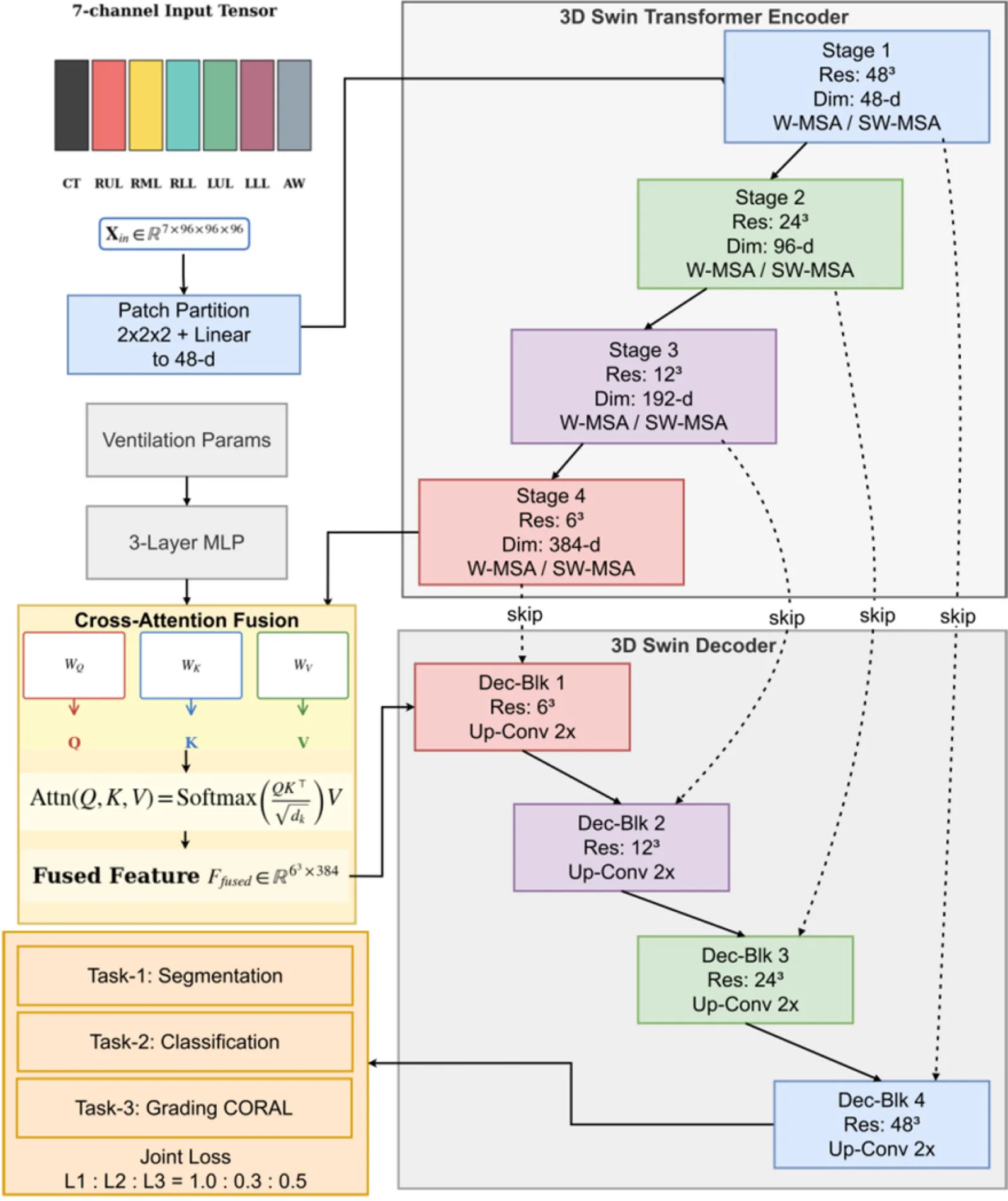
Multi-task network. AW, airway; CORAL, COnsistent RAnk Logits.

As shown in Figure 3, the second-stage multi-task network designed in this paper adopts an end-to-end architecture of “encoder—cross-modal fusion—decoder—three task heads”. The network takes a 7-channel tensor (original CT image + 5 lung lobe masks + airway mask) as input, which is divided into 2 × 2 × 2 non-overlapping sub-patches by the Patch Partition layer and linearly embedded into a 48-dimensional feature space; then it enters a four-level 3D Swin Transformer encoder. Each stage alternately realizes local window attention and cross-window information exchange through W-MSA (Window-based Multi-head Self-Attention) and SW-MSA (Shifted Window-based Multi-head Self-Attention). The feature dimension is progressively expanded from 48-d to 384-d; the spatial resolution is downsampled from 48^3^ to 6^3^; the number of multi-head attention heads is simultaneously increased from 3 to 24. At the bottleneck, the 5-dimensional ventilation parameter vector is encoded into 128-dimensional clinical features through a 3-layer MLP (Multi-Layer Perceptron). A Cross-Attention module generates queries from visual features and keys and values from clinical features, achieving deep cross-modal coupling. Residual connections ensure no loss of original visual information. The fused features enter a symmetrical 4-level Swin decoder, gradually restoring the original resolution to 96^3^ through skip connections and upsampling. Finally, three task heads branch out: the Segmentation Head outputs voxel-level probability maps of five types of signs; the Classification Head outputs multi-label classification probabilities after global average pooling; and the Grading Head implements ordered three-level severity grading based on the CORAL framework. The overall network is trained using a joint loss (weight ratio 1.0:0.3:0.5) consisting of Focal-Tversky, BCE (Binary Cross-Entropy), and CORAL, achieving collaborative learning of pixel-region-overall semantics at three levels.

#### 3.4.1 Backbone network architecture

This study uses 3D Swin UNETR (35, 36) as the backbone architecture of the multi-task network. The network input adopts a 96times96times96 three-dimensional patch. The input volume is divided into non-overlapping 2times2times2 sub-patches through the Patch Partition layer and linearly embedded into the 48-dimensional feature space. The encoder consists of four hierarchical stages, with the feature dimensions of each stage being [48,96,192,384], the spatial resolution being downsampled to $\left[48^{3},24^{3},12^{3},6^{3}\right]$, and the number of multi-head attention heads being [3,6,12,24]. Each Stage contains two Swing Transformer Blocks. Each Block consists of alternating windowed multi-head self-attention (W-MSA) and shifted windowed multi-head self-attention (SW-MSA):

$$\begin{array}{rl} {\hat{z}}^{l} & \;=W\text{-}\mathrm{MSA}(\mathrm{LN}(z^{l-1}))+z^{l\text{-}1}\\ z^{l} & \;=\mathrm{MLP}(\mathrm{LN}({\hat{z}}^{l}))+{\hat{z}}^{l}\\ {\hat{z}}^{l\;+\;1} & \;=\mathrm{SW}\text{-}\mathrm{MSA}(\mathrm{LN}(z^{l}))+z^{l}\\ z^{l\;+\;1} & \;=\mathrm{MLP}(\mathrm{LN}({\hat{z}}^{l\;+\;1}))+{\hat{z}}^{l\;+\;1} \end{array}$$ (9)

As shown in Equation 9, the window size is set to 7times7times7, which means that the self-attention calculation is performed within a local window composed of $7^{3}=343$ voxels, reducing the original global self-attention complexity of $O(N^{2})$ to $O(N\cdot M^{2})$ (where M=7 is the window side length), significantly reducing the computational cost. Adjacent windows exchange information across windows through cyclic shifts of (⌊M/2⌋,⌊M/2⌋,⌊M/2⌋), overcoming the problem of isolated receptive fields caused by fixed windows. Stages achieve a 2x downsampling of spatial resolution and a 2x expansion of feature dimensions through Patch Merging layers.

To fully utilize the engineering experience accumulated by nnU-Net in medical image segmentation tasks, this study transplants its self-configuration mechanism to the Swin UNETR framework: (1) Patch-size adaptation—the nnU-Net-inspired configuration procedure was used to evaluate feasible patch dimensions under the available GPU memory constraint, and a fixed patch size of 96 × 96 × 96 voxels was ultimately selected and used consistently for all training, validation, and testing experiments; (2) Batch size adaptation—automatically setting it to 2 based on the patch size and the single-card memory limit; (3) Pooling operand adaptation—automatically determining the number of encoder pooling layers based on the input resolution and target downsampling ratio; (4) Spacing perception—resampling all inputs to isotropic $1\;\mathrm{times}1\;\mathrm{times}1\;mm^{3}$ to ensure spatial consistency of the receptive field of the 3D convolutional kernel.

#### 3.4.2 Design of three task headers

The segmentation head is directly connected to the final output feature map $F_{dec}\in R^{48\times 96^{3}}$ of the decoder. It generates voxel-level probability maps for five sign categories through two layers of 3D transposed convolution and a Sigmoid activation function:

$${\hat{Y}}_{seg}=\sigma (\mathrm{ConvT}3D_{2}(\mathrm{ReLU}(\mathrm{ConvT}3D_{1}(F_{dec}))))\in {\left[0,1\right]}^{C\;\times H\;\times W\;\times D}$$ (10)

As shown in Equation 10, C=5 (GGO, Real Variation, Nodule, Cavity, Pleural Effusion), and σ(⋅) is the Sigmoid function to support multi-label overlapping prediction.

The classification head starts from the encoder bottleneck feature $F_{v}\in R^{6^{3}\;\times 384}$, aggregates spatial information through global average pooling to obtain a 384-dimensional image-level feature vector, and then outputs the existence probability of 5 types of features through two fully connected layers:

$$\begin{array}{rl} f_{GAP} & =\frac{1}{H^{\prime }W^{\prime }D^{\prime }}\sum_{i,j,k}F_{v}(i,j,k),\\ {\hat{Y}}_{cls} & =\sigma (W_{2}\cdot \mathrm{GELU}(W_{1}f_{GAP}+b_{1})+b_{2}) \end{array}$$ (11)

In the Equation 11, $W_{1}\in R^{128\times 384}$ and $W_{2}\in R^{5\times 128}$ are learnable weights. Sigmoid activation makes the output of each category independent and can support multi-label classification.

The three levels of VAP severity, “light—medium—severe,” are essentially ordered categories. The cost of misclassification between adjacent categories is much lower than that of cross-level misclassification. Traditional Softmax multi-class classification cannot explicitly encode this ordinal relationship. Therefore, this study adopts the CORAL framework to achieve ordered classification. The core idea is to decompose the K-class ordered classification problem into K-1 binary sub-problems. Each sub-problem judges whether “the sample belongs to level *k* or above”:

$$P(y\;>k|x)=\sigma (w^{⊤}f_{GAP}\text{-}b_{k}),k=1,2,\;\mathrm{ldots},K\text{-}1$$ (12)

As shown in Equation 12, all K-1 binary classifiers share the same weight vector w, only the bias term $b_{k}$ is different.

#### 3.4.3 Multimodal fusion module

Clinical assessment of VAP naturally possesses multimodal attributes: CT provides structural imaging features, while ventilation parameters reflect respiratory physiological status, and the two complement each other. This study introduces a multimodal fusion module based on the Cross-Attention mechanism at the encoder bottleneck.

This study selects five ventilation parameters closely related to the severity of VAP to construct a clinical feature vector $x_{c}=[\mathrm{PEEP},P/F,T_{vent},V_{T},\mathrm{Fi}O_{2}{]}^{⊤}\in R^{5}$. To prevent temporal information leakage, all clinical variables were restricted to information available at or before the CT examination. PEEP, tidal volume, and FiO₂ were defined as the values recorded closest to CT acquisition within the preceding 2 h, while the PaO₂/FiO₂ ratio was calculated from the arterial blood gas measurement closest to CT within the preceding 6 h. Ventilation duration was defined as the elapsed time from initiation of invasive mechanical ventilation to CT acquisition, rather than the total subsequent ventilation duration. No post-CT measurements or outcomes were used as model inputs. Considering the differences in the numerical dimensions of each parameter (e.g., PEEP is in cmH₂O, dimension 5–20; ventilation duration is in hours, dimension 48–720), each dimension is first normalized to the [0,1] interval using Min-Max, and then encoded into 128-dimensional semantic features by 3 layers of MLP, as shown in following Equation 13:

$$f_{c}=W_{3}\cdot \mathrm{GELU}(W_{2}\cdot \mathrm{GELU}(W_{1}x_{c}+b_{1})+b_{2})+b_{3}\in R^{128}$$ (13)

The hidden layer dimensions are 5→32→64→128, and Dropout (p=0.1) is inserted between each layer to prevent overfitting.

Let the visual features output by the encoder bottleneck be $F_{v}\in R^{N_{v}\;\times d_{v}}$ ($N_{v}=6^{3}=216$ is the number of spatial locations, and $d_{v}=384$ is the feature dimension), and the clinical features be $f_{c}\in R^{128}$. The fusion module uses visual features as the Query and clinical features as the Key and Value. By scaling the dot product attention, the visual features can selectively “query” relevant information in the clinical parameters:

$$Q=F_{v}W_{Q},K=f_{c}W_{K},V=f_{c}W_{V}$$ (14)

$$F_{\;fused}=\mathrm{Softmax}\left(\frac{QK^{⊤}}{\sqrt{d_{k}}}\right)V+F_{v}$$ (15)

In Equations 14–15, $W_{Q}\in R^{d_{v}\;\times d_{k}}$ and $W_{K},W_{V}\in R^{128\times d_{k}}$ are the learnable projection matrices; $d_{k}=64$ is the internal dimension of the attention head; the residual term $+F_{v}$ ensures that the features do not lose the original visual information after fusion.

### 3.5 Loss function and training strategy

#### 3.5.1 Focal-Tversky hybrid segmentation loss

Tversky Loss extends the symmetric structure of the traditional Dice Loss by introducing asymmetric coefficients β and 1-β to control the penalty weights for false negatives and false positives, respectively:

$$L_{Tversky}=1\text{-}{\left(\frac{\sum_{i}p_{i}g_{i}+\varepsilon }{\sum_{i}p_{i}g_{i}+\beta \sum_{i}(1\text{-}p_{i})g_{i}+(1\text{-}\beta )\sum_{i}p_{i}(1\text{-}g_{i})+\varepsilon }\right)}^{1/\gamma_{t}}$$ (16)

In the Equation 16, $p_{i}$ is the predicted probability of the i-th voxel; $g_{i}\in \{0,1\}$ is the corresponding gold standard label; β=0.7 makes the penalty weight for false negatives 2.33 times that for false positives; $\gamma_{t}=0.75\;\mathrm{lt}1$ is an exponential factor that further amplifies the gradient contribution of hard-to-separate samples; $\varepsilon =10^{\text{-}6}$ is a smoothing term to prevent division by zero.

Focal Loss reduces the loss weights of easily separable samples by modulating a factor $(1p_{t}{)}^{\gamma }$, forcing the model to focus on boundary voxels that are difficult to classify:

$$L_{Focal}=\text{-}\frac{1}{N}\sum_{i}\alpha_{t}{\left(1\text{-}p_{t}\right)}^{\gamma }\log(p_{t})$$ (17)

In the Equation 17, $p_{t}=p_{i}$ if $g_{i}=1$ else $1\text{-}p_{i}$, focusing factor γ=2, class balance weight $\alpha_{t}=0.75$ (foreground) or 0.25 (background). The final segmentation loss is a weighted combination of the two, as shown in Equation 18:

$$L_{seg}=\alpha \cdot L_{Tversky}+(1\text{-}\alpha )\cdot L_{Focal},\alpha =0.6$$ (18)

#### 3.5.2 Joint loss for multiple tasks

The losses of the three task heads are combined into a joint loss function by weighted summation. The classification head uses standard binary cross-entropy to support multi-label prediction, as shown in Equation 19:

$$L_{cls}=\text{-}\frac{1}{C}\sum_{c=1}^{C}\left[y_{c}\log{\hat{y}}_{c}+(1\text{-}y_{c})\log(1\text{-}{\hat{y}}_{c})\right]$$ (19)

The grader uses equal-weighted summation of binary cross-entropy as the loss under the CORAL framework, as shown in Equation 20:

$$L_{grade}=\text{-}\sum_{k=1}^{K-1}\left[1[y\;>k]\log P(y\;>k)+1[y\leq k]\log(1\text{-}P(y\;\mathrm{gt}k))\right]$$ (20)

The total loss combines the three tasks, with the loss weights set to 1.0:0.3:0.5. These weights were selected by grid search using only the training and internal-validation cohorts. The external test cohort was not used for loss-weight selection, hyperparameter tuning, early stopping, or model selection. The selected weights were fixed before external testing and remained unchanged throughout all external-validation analyses, as shonw in Equation 21:

$$L_{total}=\lambda_{1}L_{seg}+\lambda_{2}L_{cls}+\lambda_{3}L_{grade}$$ (21)

#### 3.5.3 Training hyperparameters and implementation details

All training experiments are conducted on two NVIDIA A100 80GB GPUs (Graphics Processing) The training is conducted on the server of Unit (CUDA 12.1, PyTorch 2.1). The optimizer used is AdamW ($\beta_{1}=0.9$, $\beta_{2}=0.999$, weight decay $=10^{\text{-}5}$), with an initial learning rate $lr_{init}=10^{\text{-}4}$. After 5 epochs of linear warm-up, the scheduler switched to CosineAnnealingLR:

$$\mathrm{lr}(t)=lr_{\min}+\frac{1}{2}(lr_{init}\text{-}lr_{\min})\left(1+\cos\left(\frac{t\text{-}t_{warm}}{T\text{-}t_{warm}}\pi \right)\right)$$ (22)

In the Equation 22, $lr_{\min}=10^{\text{-}6}$; T=400 epochs is the total number of training rounds; $t_{warm}=5$. All experiments used a fixed 3D patch size of 96 × 96 × 96 voxels. The batch size was set to 2 because of the memory requirements of the 96^3^ patches, and gradient accumulation (accumulation = 4) yielded an effective batch size of 8. To accelerate training convergence and save memory, automatic mixed precision training is enabled.

### 3.6 Formulation of the algorithmic radiological burden grading criteria

This study defines an algorithmic radiological burden grading scheme to quantitatively summarize the extent and distribution of VAP-related CT abnormalities. The scheme is based on the lesion-to-lung volume ratio ($V_{ratio}$), the number of involved pulmonary lobes ($N_{lobe}$), and the presence of high-risk radiological findings such as cavitation. These variables characterize complementary aspects of radiological disease burden, including lesion extent, anatomical dissemination, and destructive pulmonary involvement.

For construction of the reference grading targets, $V_{ratio}$ and $N_{lobe}$ were calculated from the radiologist-adjudicated lesion masks together with the anatomical lung-lobe masks. The resulting categories were therefore derived deterministically from quantitative imaging characteristics rather than independently assigned by radiologists. Accordingly, these categories are referred to throughout the revised manuscript as mild, moderate, and severe radiological burden grades, rather than independently validated clinical VAP severity grades.

The operational thresholds were informed by previously reported CT-based pneumonia burden scoring approaches, including the Fleischner Society-related pneumonia assessment framework, the modified Brixia score, and the COVID-19 CT Severity Score (37, 38). Mild radiological burden was defined as $V_{ratio}<15\text{\%}$, $N_{lobe}\leq 2$, and absence of cavitation; moderate burden was defined as $15\text{\%}\leq V_{ratio}<35\text{\%}\;$ or involvement of three to four lobes; and severe burden was defined as $V_{ratio}\geq 35\text{\%}$, involvement of all five lobes, or the presence of cavitation. These thresholds constitute an operational imaging-based stratification rule and should not be interpreted as clinically validated VAP severity cutoffs.

The 15% and 35% cutoffs were adopted as pragmatic radiological-burden thresholds rather than established VAP-specific clinical cutoffs. In the VAP setting, volumetric involvement was combined with lobar dissemination and cavitation because lesion extent alone may not adequately represent anatomically diffuse or destructive pulmonary disease. Accordingly, the clinical relevance of the proposed stratification was evaluated against established critical-care indices and 28-day mortality, rather than inferred solely from pneumonia scoring systems developed in other disease contexts.

## 4 Experiments

### 4.1 Dataset splitting

This research utilized data collected from patients with VAP who were admitted to a tertiary general hospital's Intensive Care Unit during the time frame of January 2020 through June 2024. A total of 512 Chest CT images were obtained after strict adherence to inclusion and exclusion criteria. All 512 enrolled cases had clinically diagnosed VAP according to the predefined inclusion criteria. No non-VAP control population was included; therefore, diagnostic discrimination between VAP and alternative pulmonary conditions was outside the scope of the present study. Each case contained complete records of mechanical ventilation parameters, pathogen culture results, and ICU clinical outcomes. The dataset was divided using a stratified random partitioning approach into 3 randomly selected subsets of patients without overlap in the following ratio: training dataset consisting of 358 cases for training the model parameter, internal validation dataset consisting of 77 cases for tuning hyperparameters and judging early stopping, and external validation dataset consisting of 77 cases collected independently from a collaborating tertiary care facility, using composite data or CT scanners different than those used by the original facility, relative to the first two datasets. The stratification variables included consideration of severity level, primary pathogen type, and age group (<60, 60–70, >70), in order to provide a uniform distribution of the principal clinical characteristics of patients in the three datasets and to minimize the systematic influence of data partitioning bias on model evaluation.

To reduce overfitting on the relatively small VAP cohort, source-domain pretraining was performed before VAP-specific fine-tuning. For COVID-19-CT-Seg, the Swin encoder, decoder, and segmentation head were pretrained for 100 epochs using the available voxel-level ground-glass opacity and consolidation annotations. These two source labels were directly mapped to the corresponding GGO and consolidation channels in the VAP segmentation task, whereas the centrilobular-nodule, cavitation, and pleural-effusion channels had no source-domain counterparts and were initialized randomly. MosMedData was subsequently used for 50 epochs of ordinal representation pretraining using its original CT-0 to CT-4 severity labels and a temporary CORAL grading head. Because the MosMedData grades and the proposed VAP radiological-burden categories have different clinical definitions, no direct grade-to-grade relabeling was performed; only the learned feature representations were transferred, and the final VAP grading head was reinitialized. The multi-label classification head, clinical MLP, and Cross-Attention module were also initialized from scratch because equivalent labels and ventilation variables were unavailable in the public datasets.

For VAP fine-tuning, the transferred Swin encoder and decoder were initially frozen for the first 10 epochs while the newly initialized VAP-specific task heads and multimodal fusion layers were optimized. All second-stage network parameters were then unfrozen and jointly fine-tuned for 100 additional epochs using the multi-task objective described in Section 2.5. The learning rate for transferred parameters was set to $5\times 10^{-5}$, whereas newly initialized layers used $1\times 10^{-4}$. TotalSegmentator remained frozen throughout training and was used only for anatomical prior extraction.

The pre-training data source includes two public pneumonia CT datasets: the MosMedData dataset (with CT grade 0 through 4) as a critical transfer prior for grading and the COVID-19-CT-Seg dataset with voxel-level GGO (ground-glass opacity) and gold standard for solid state. Although both datasets are based on COVID-19 etiologies (not VAP), there is a large amount of overlap in radiological features (GGO, solid state, cobblestone sign) between both datasets and VAP, so transfer learning can likely work in the medical semantic space.

To demonstrate the consistency of the distribution of key features such as demographic baseline, mechanical ventilation parameters, clinical severity scores, and etiological composition among the training set, internal validation set, and external test set, Table 1 lists a detailed comparison of the clinical baseline features of the three subsets.

**Table 1:** Detailed composition and baseline clinical characteristics of the dataset.

Characteristics	Training set (*n* = 358)	Internal validation (*n* = 77)	External test (*n* = 77)	*P* value
Demographics				
Age (years, mean ± SD)	64.8 ± 13.2	65.3 ± 12.8	66.1 ± 14.0	0.712^a^
Male/Female (*n*, %)	218/140 (60.9%/39.1%)	48/29 (62.3%/37.7%)	47/30 (61.0%/39.0%)	0.965^b^
BMI (kg/m^2^, mean ± SD)	23.6 ± 4.1	23.9 ± 3.8	23.4 ± 4.3	0.683^a^
Mechanical ventilation				
Ventilation duration before CT (h, median [IQR])	168 [96, 312]	180 [104, 288]	192 [112, 336]	0.418^c^
PEEP (cmH₂O, mean ± SD)	8.4 ± 3.2	8.7 ± 3.0	8.2 ± 3.5	0.592^a^
PaO₂/FiO₂ ratio (mean ± SD)	192.5 ± 78.6	188.3 ± 82.1	196.7 ± 75.4	0.784^a^
Severity Scores				
APACHE II (mean ± SD)	18.2 ± 6.7	17.9 ± 6.9	18.6 ± 7.1	0.801^a^
SOFA (mean ± SD)	7.8 ± 3.4	7.6 ± 3.5	8.0 ± 3.6	0.756^a^
CPIS (mean ± SD)	6.9 ± 1.8	7.1 ± 1.7	6.8 ± 1.9	0.624^a^
Predominant pathogens (*n*, %)				0.872^b^
*Pseudomonas aeruginosa*	108 (30.2%)	23 (29.9%)	24 (31.2%)	—
*Acinetobacter baumannii*	89 (24.9%)	20 (26.0%)	19 (24.7%)	—
*Staphylococcus aureus* (incl. MRSA)	72 (20.1%)	15 (19.5%)	14 (18.2%)	—
*Klebsiella pneumoniae*	54 (15.1%)	12 (15.6%)	13 (16.9%)	—
Others/Polymicrobial	35 (9.8%)	7 (9.1%)	7 (9.1%)	—
Severity distribution (*n*, %)				0.983^b^
Mild (Grade I)	125 (34.9%)	27 (35.1%)	26 (33.8%)	—
Moderate (Grade II)	143 (39.9%)	31 (40.3%)	31 (40.3%)	—
Severe (Grade III)	90 (25.1%)	19 (24.7%)	20 (26.0%)	—
Clinical outcomes				
28-day mortality (*n*, %)	71 (19.8%)	15 (19.5%)	17 (22.1%)	0.891^b^
ICU length of stay (days, median [IQR])	18 [12, 28]	19 [11, 30]	20 [13, 31]	0.547^c^

All *P* > 0.05 indicate no statistically significant difference among the three subsets, confirming balanced distribution.

IQR, interquartile range; PEEP, positive end-expiratory pressure; APACHE II, Acute Physiology and Chronic Health Evaluation II; SOFA, Sequential Organ Failure Assessment; CPIS, Clinical Pulmonary Infection Score; MRSA, methicillin-resistant *Staphylococcus aureus*; BMI, body mass index. All participants included in the three subsets had clinically diagnosed VAP; no non-VAP comparator group was included in the present cohort.

Data are presented as mean ± SD (Standard Deviation) for normally distributed continuous variables, median [IQR] for skewed variables, and *n* (%) for categorical variables. *P* values were computed across the three subsets using:

a one-way ANOVA (Analysis of Variance).

b chi-square test.

c Kruskal–Wallis test.

### 4.2 Evaluation index system

The output of the segmentation task is a binary mask obtained by thresholding the voxel-level probability map of the lesion. Its quality assessment needs to consider both regional overlap accuracy and boundary localization accuracy. This study uses four complementary indicators for comprehensive quantification (39–41): Dice Similarity Coefficient (DSC), 95th percentile Hausdorff Distance (HD95), Average Surface Distance (ASD), and Volume Relative Error (VRE).

DSC is the most widely used region overlap metric in the field of medical image segmentation (42, 43). It reflects the voxel-level consistency between the predicted mask and the gold standard, and its value ranges from [0, 1]. The larger the value, the higher the segmentation accuracy:

$$\mathrm{DSC}(P,G)=\frac{2|P\cap G|}{|P|+|G|}=\frac{2\sum_{i}p_{i}g_{i}}{\sum_{i}p_{i}+\sum_{i}g_{i}}$$ (23)

In the Equation 23, P and *G* are the voxel sets of the predicted mask and the gold standard mask, respectively, and $p_{i}\in \{0,1\}$ and $g_{i}\in \{0,1\}$ are the corresponding binary labels.

The traditional Hausdorff distance measures the maximum surface deviation between two point sets, but it is extremely sensitive to individual outliers (44, 45). HD95 effectively suppresses the influence of outliers by taking the 95th percentile value, and more robustly reflects the overall boundary error. ASD characterizes the average shortest distance from each point on the predicted boundary to the gold standard boundary, focusing more on the overall average error level of the boundary compared to HD95:

$$\mathrm{ASD}(P,G)=\frac{1}{|S_{P}|+|S_{G}|}\left(\sum_{\;p\in S_{P}}d(p,S_{G})+\sum_{g\in S_{G}}d(g,S_{P})\right)$$ (24)

In the Equation 24, $S_{P}$ and $S_{G}$ are the surface voxel sets of *P* and *G*, respectively. ASD is expressed in mm; the smaller the value, the finer the segmentation boundary.

VRE is used to assess the quantitative consistency between the predicted lesion volume and the gold standard volume, which is crucial for subsequent volume-based severity grading:

$$\mathrm{VRE}(P,G)=\frac{|V_{P}\text{-}V_{G}|}{V_{G}}\;\times 100\text{\%}$$ (25)

In the Equation 25, $V_{P}=\sum_{i}\;p_{i}\cdot v_{voxel}$ and $V_{G}=\sum_{i}g_{i}\cdot v_{voxel}$ are the physical volumes of the predicted and gold standard lesions, respectively.

The classification task uses a multi-label approach to determine the presence of five radiological findings—GGO, consolidation, centrilobular nodules, cavitation, and pleural effusion—in each CT examination. These findings may occur in VAP but are not considered specific to VAP (46, 47). The core challenge lies in the uneven distribution of samples for each type of sign. This study uses the Area Under the ROC Curve (AUC) as the main evaluation index because AUC is insensitive to class imbalance and can comprehensively reflect the model's discriminative ability at various thresholds, as shown in following Equation 26:

$$\mathrm{AU}C_{c}=\int_{0}^{1}\mathrm{TP}R_{c}(t)d(\mathrm{FP}R_{c}(t))=\int_{0}^{1}\mathrm{TP}R_{c}{(\mathrm{FPR}}_{c}^{\text{-}1}(u))\mathrm{du}$$ (26)

Accordingly, the AUC reported for this task quantifies the model's ability to discriminate the presence versus absence of each radiological sign within the clinically diagnosed VAP cohort. It is not a diagnostic AUC for distinguishing VAP from non-VAP patients.

This study uses the weighted Cohen’s Kappa ($\kappa_{w}$) coefficient as the core evaluation index for the classification task, and introduces a misclassification distance weight to impose a heavier penalty on cross-level misclassification:

$$\kappa_{w}=1\text{-}\frac{\sum_{i\;=\;1}^{K}\sum_{\;j\;=\;1}^{K}w_{ij}O_{ij}}{\sum_{i=1}^{K}\sum_{\;j=1}^{K}w_{ij}E_{ij}}$$ (27)

In the Equation 27, K=3 is the number of classification categories; $O_{ij}$ is the observation confusion matrix (the number of samples predicted by the model as level *i* and the gold standard as level j); $E_{ij}$ is the expected frequency under the assumption that the prediction and the gold standard are independent:

$$E_{ij}=\frac{\left(\sum_{k}O_{ik})(\sum_{k}O_{kj}\right)}{N}$$ (28)

In the Equation 28, N is the total number of samples. The weight matrix $w_{ij}$ uses a quadratic weight to match the grade difference characteristics of ordered classification:

$$w_{ij}=\frac{(i-j{)}^{2}}{(K-1{)}^{2}}$$ (29)

According to the Equation 29, under this weight definition, the weight of same-level prediction (i=j) is 0 and is not included in the error, the weight of adjacent-level misjudgment (∣i−j∣=1) is $1/(K\text{-}1{)}^{2}=0.25$, and the weight of cross-level misjudgment (∣i−j∣=2) is 1.0. The penalty for cross-level error is 4 times that of adjacent error, which is more in line with the clinical understanding of the cost of misjudgment of severity. The value range of $\kappa_{w}$ is [-1,1], where $\kappa_{w}=1$ indicates perfect consistency, $\kappa_{w}=0$ indicates only random consistency, and $\kappa_{w}\;<0$ indicates that the consistency is lower than the random expectation. In this study, weighted Cohen’s κ is used to quantify agreement between the predicted ordinal category and the predefined reference category. A value of κ≥0.80 is interpreted as strong agreement; however, it should not be regarded as independent evidence of clinical usability.

In the present study, weighted Cohen's *κ* quantifies agreement between the model-predicted ordinal radiological burden category and the algorithmically generated reference category. It therefore evaluates the model's ability to reproduce the predefined radiological burden stratification and should not be interpreted as agreement with an independently established clinician-derived VAP severity grade.

### 4.3 Comparative experiment design

To objectively evaluate the performance advantages of the proposed method, this study selected five representative deep learning models in the field of medical image segmentation as baselines: 3D U-Net, representing the classic convolutional encoder-decoder architecture; V-Net, which introduces residual connections and Dice Loss to optimize volume segmentation; nnU-Net, which achieves strong baseline performance on multiple benchmarks through a self-configuration mechanism; 3D Swin UNETR (the original version, without the proposed multimodal fusion and multi-task extensions) representing the Transformer-like architecture; and TransUNet-3D, which integrates the hybrid feature extraction capabilities of CNN (Convolutional Neural Network) and Transformer. All baseline models were trained fairly under the same data partitioning, preprocessing procedures, and training hyperparameters, retaining only the differences in their original network structures.

To quantify the independent contribution of each core module to the final performance, this study designed a systematic ablation experiment: Anatomical priors were removed, and the 7-channel input was reduced to a single-channel CT input to examine the guiding role of the lung lobe mask in lesion localization; ventilation parameter fusion was removed, and the Cross-Attention module was removed, retaining only pure visual features for segmentation and grading; Focal-Tversky loss was removed, and the segmentation loss was replaced with the standard Dice Loss to evaluate the improvement effect of the hybrid loss function on small lesions and class imbalance scenarios; single-task and multi-task comparisons were conducted, training independent segmentation, classification, and grading networks respectively to examine the regularization effect and inter-task synergistic gains of multi-task joint learning; the backbone network was replaced, with the encoder replaced with 3D ResNet-50 (Residual Network with 50 layers) and 3D ConvNeXt (Convolutional Network of the Next Generation) respectively, to verify the architectural advantages of the Swin Transformer in global feature modeling.

The model's cross-center and cross-device generalization ability is a key dimension for evaluating its clinical deployment potential. This study validated generalization on two levels: First, a blind test was conducted on an external test set (77 cases) from an independent collaborating hospital. The CT equipment model and scanning technician team of this center were completely different from those of the first center, which could realistically simulate the domain shift challenge in clinical deployment scenarios. Second, within the external test set, the patients were divided into three subgroups according to CT equipment manufacturers: GE (28 cases), Siemens (26 cases), and Philips (23 cases). One-way ANOVA was used to test whether there were significant differences in the Dice coefficient among the three manufacturers, in order to quantify the robustness of the model to equipment heterogeneity.

## 5 Results

### 5.1 Lesion segmentation performance results

To quantitatively evaluate the performance advantages of the model in this paper on the lesion segmentation task, Table 2 summarizes the comprehensive comparison results of the segmentation accuracy, parameter size and inference efficiency of mainstream methods on the internal validation set.

**Table 2:** Comparison of lesion segmentation performance across seven methods on internal validation set.

Method	Dice ↑	HD95 (mm) ↓	ASD (mm) ↓	VRE (%) ↓	Params (M)	Inference time (s)
3D U-Net	0.781 ± 0.052***	13.45 ± 4.21***	3.18 ± 1.05***	18.62 ± 6.31***	19.1	1.2
V-Net	0.795 ± 0.048***	12.76 ± 3.95***	2.94 ± 0.97***	16.85 ± 5.92***	22.6	1.4
nnU-Net	0.812 ± 0.044**	11.23 ± 3.68**	2.61 ± 0.88**	14.27 ± 5.46**	30.8	1.6
3D Swin UNETR (vanilla)	0.823 ± 0.041**	10.58 ± 3.32**	2.45 ± 0.81**	13.18 ± 4.93**	51.2	1.7
TransUNet-3D	0.819 ± 0.043**	10.91 ± 3.47**	2.52 ± 0.84**	13.74 ± 5.12**	67.5	2.1
Ours *(w/o multi-modal)*	0.831 ± 0.039*	9.74 ± 2.96*	2.18 ± 0.73*	11.89 ± 4.35*	58.4	1.7
Ours *(full model)*	**0.847** **±** **0.035**	**8.32** **±** **2.54**	**1.76** **±** **0.61**	**9.82** **±** **3.78**	62.7	1.8

Values are presented as mean ± SD across all internal validation cases. ↑ indicates higher is better. ↓ indicates lower is better.

HD95, 95th percentile Hausdorff distance; ASD, average surface distance; VRE, volume relative error.

Bold denotes the best result. Statistical significance vs. the proposed full model is computed by paired Student's t-test:

* *P* < 0.05.

** *P* < 0.01.

*** *P* < 0.001.

As shown in Table 2, the complete model in this paper achieves the best performance in all four segmentation accuracy metrics: the Dice coefficient reaches 0.847 ± 0.035, an improvement of 8.5% compared to the 3D U-Net baseline (0.781) and 4.3% compared to the strong baseline nnU-Net (0.812); HD95 is reduced to 8.32 mm, a decrease of 38.1% compared to 3D U-Net, indicating a significant improvement in boundary localization accuracy; ASD and VRE are 1.76 mm and 9.82% respectively, both the lowest levels among all methods, demonstrating the ability to accurately estimate lesion volume, which is crucial for subsequent severity grading. Paired t-tests show that the differences between the model in this paper and all baseline methods are statistically significant (*P* < 0.05), especially reaching a highly significant level of *P* < 0.001 compared to the 3D U-Net and V-Net methods. In terms of efficiency, the model in this paper has 62.7 M parameters and an inference time of 1.8 s, proving that the performance improvement is not at the expense of huge computational overhead. Ablation comparison (w/o multimodal model Dice = 0.831) validates the contribution of ventilation parameter fusion, highlighting the effectiveness of the multimodal architecture design.

To visually verify the advantages of thye proposed model over the classic baseline method in segmentation visual effects on VAP cases of different severities, Figure 4 selects three representative cases (mild, moderate, and severe) and displays the segmentation results of the original CT scan, the radiology gold standard, and the three methods in parallel.

**Figure 4:**
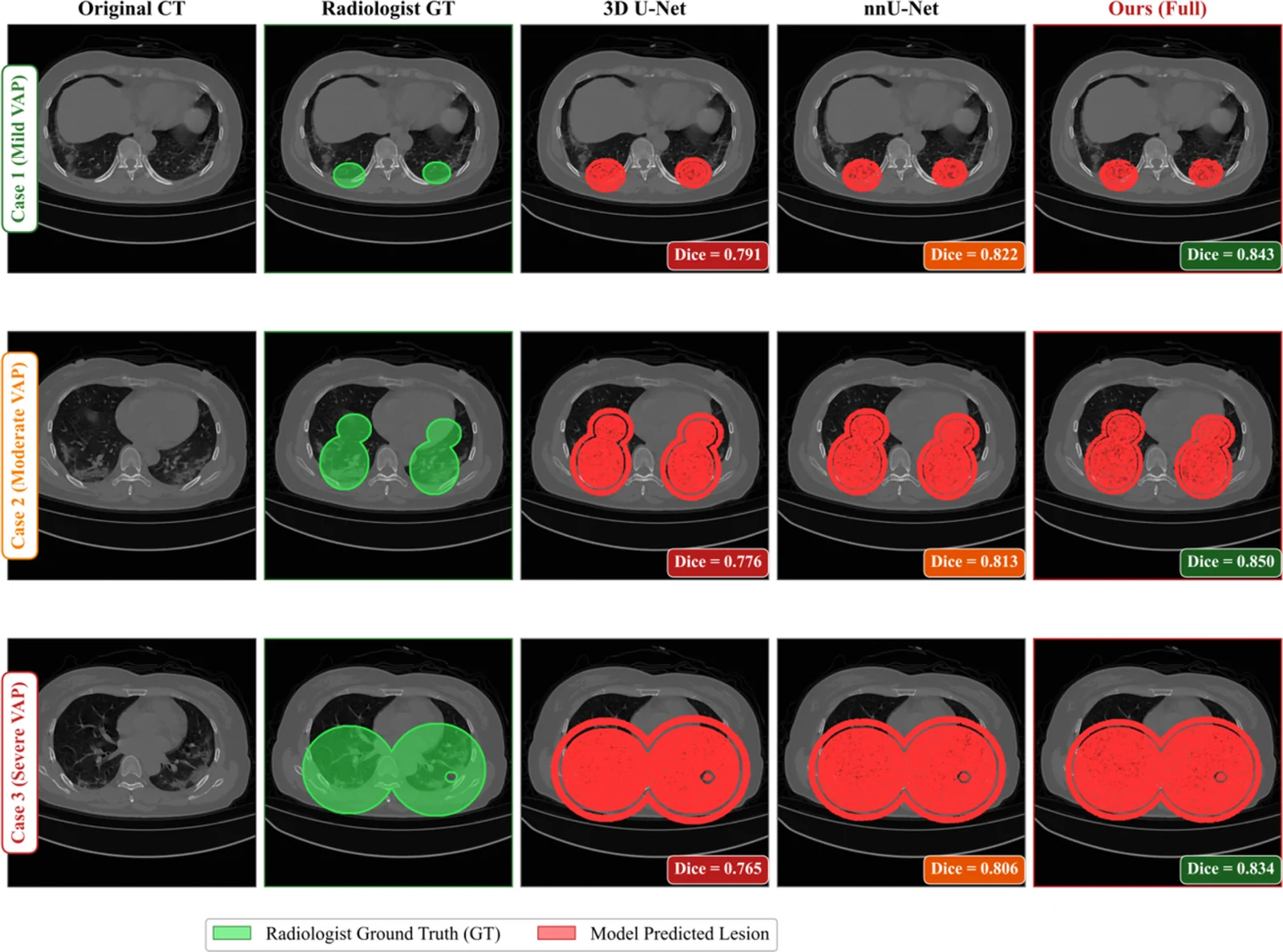
Visual comparison of segmentation results in typical cases.

As shown in Figure 4, the visual comparison of segmentation results from three representative cases (mild, moderate, and severe VAP) using multiple methods intuitively demonstrates the significant advantages of the proposed model compared to the baseline. The mild VAP case (Case 1) presented two symmetrical small ground-glass opacities in the gravity-dependent areas of both lung dorsal regions (a typical early manifestation of VAP in supine patients). 3D U-Net, due to its limited receptive field, failed to capture small-scale lesions adequately, resulting in rough boundaries (Dice = 0.791). nnU-Net showed some improvement but still exhibited slight boundary expansion (Dice = 0.822). The proposed model, with the aid of anatomical prior guidance and Cross-Attention fusion, accurately located bilateral subtle lesions, and the boundaries closely matched the gold standard (Dice = 0.843). The moderate VAP case (Case 2) presented with multifocal lesions in both lungs. 3D U-Net exhibited significant boundary expansion and incorrect merging of connected components in the multifocal scenario (Dice = 0.776), followed by nnU-Net (Dice = 0.813). The proposed model, with its multimodal fusion mechanism, demonstrated stronger multifocal lesion separation capability (Dice = 0.850). The severe VAP case (Case 3) presented with diffuse lesions in both lungs combined with necrotic cavitation. Due to the large lesion area, the Dice values of all methods decreased slightly (U-Net = 0.765, nnU-Net = 0.806). The proposed model, utilizing the high sensitivity of Focal-Tversky loss to small targets, accurately separated the cavitary structures (Dice = 0.834). The visual effects and quantitative Dice indicators of the three cases collectively confirm the reliability and clinical applicability of the results summarized in Table 2.

### 5.2 Multi-label classification results of radiological signs

To comprehensively evaluate the discriminative ability and confusion patterns of the proposed model for the five radiological findings, Figure 5 presents the multi-label classification performance using ROC curves and the normalized confusion matrix.

**Figure 5:**
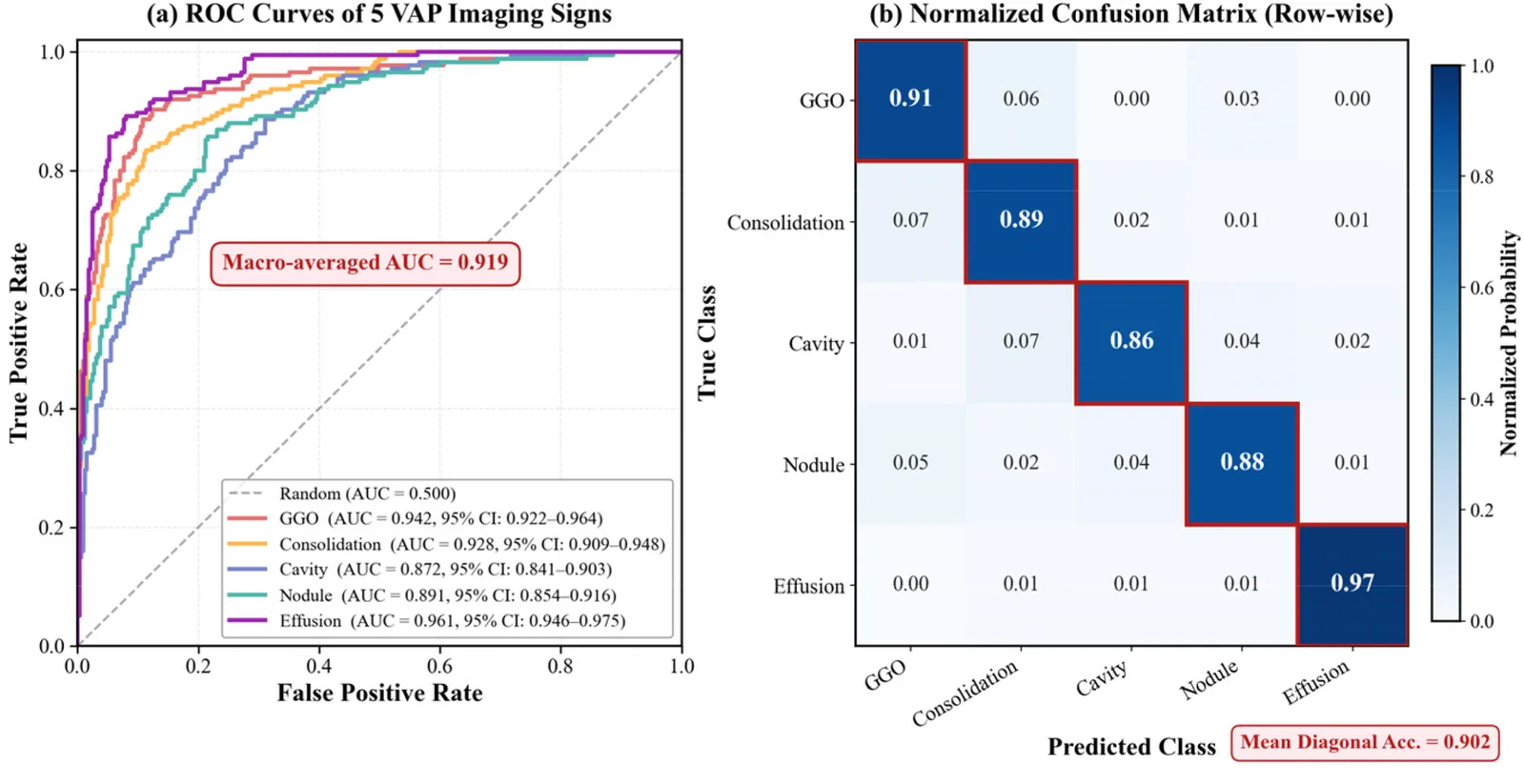
ROC curves and confusion matrix for multi-label classification of signs. **(a)** ROC curves of 5 VAP imaging signs; **(b)** normalized confusion matrix (row-wise).

As shown in Figure 5a, the ROC curves of the model in this paper were significantly far from the random classification diagonal (AUC = 0.500) for all five radiological findings, demonstrating excellent multi-label discrimination ability. Specifically, pleural effusion achieved the highest AUC = 0.961 (95% CI: 0.946–0.975), thanks to its clear imaging boundary formed by the high density difference with the lung parenchyma; ground-glass opacity AUC = 0.942 and consolidation AUC = 0.928, both being the most common and morphologically distinctive signs of VAP, with recognition accuracy close to the upper limit; centrilobular nodules AUC = 0.891, slightly lower than the previous three due to the sparse features of small-scale targets; cavitation AUC = 0.872 was the lowest among the five types, mainly because the gas-necrosis boundary inside the cavity is similar in gray level to the background of the lung parenchyma. The macro-average AUC across the five radiological signs was 0.919, demonstrating strong sign-level discrimination within the clinically diagnosed VAP cohort. This result reflects radiological-sign classification performance and does not represent diagnostic discrimination between VAP and non-VAP conditions.

The normalized confusion matrix in Figure 5b further reveals the confusion patterns and misclassification rules among the features in the model. The correct recognition rates for the five diagonal features are GGO = 0.91, consolidation = 0.89, cavity = 0.86, nodule = 0.88, and pleural effusion = 0.97, with an average diagonal accuracy of 0.902. The most significant confusion occurs between GGO and consolidation (GGO misclassified as consolidation rate 0.06, reverse misclassification rate 0.07), consistent with the clinical radiographic progression that “GGO is a precursor to early consolidation.” There is a 0.07 confusion rate between cavity and consolidation, because cavities are usually nested within consolidation areas. There is a 0.05 confusion rate between nodule and GGO, as small-scale GGO nodules are visually similar. The confusion rate between pleural effusion and the other four features is ≤0.01, reflecting the high independence of their radiographic features.

### 5.3 Radiological burden grading results

To comprehensively evaluate the grading accuracy, inter-method advantages, and correlation between grading results and clinical critical care scores of the model presented in this paper on the three-level VAP severity grading task, Figure 6 presents the grading performance from four complementary dimensions.

**Figure 6:**
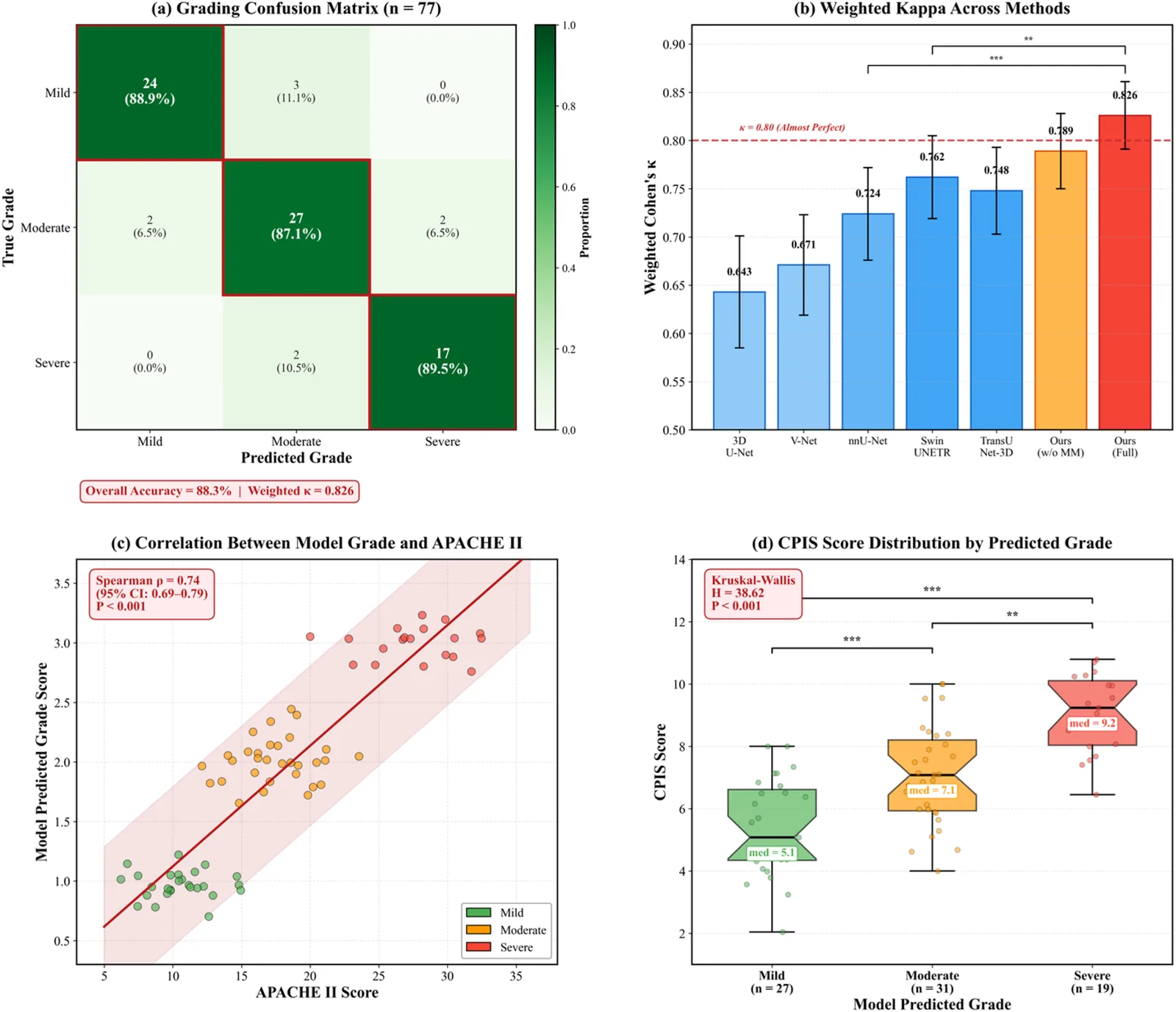
Severity grading results. w/o MM, w/o multi-modal. **(a)** Grading confusion matrix (*n* = 77); **(b)** weighted kappa across methods; **(c)** correlation between model grade and APACHE; **(d)** CPIS score distribution by predicted grade.

As shown in Figure 6a, the three-level radiological burden confusion matrix on the internal validation set demonstrated an overall accuracy of 88.3%, with class-specific accuracies of 88.9% for mild burden, 87.1% for moderate burden, and 89.5% for severe burden. The weighted κ was 0.826, indicating strong agreement between the model predictions and the predefined algorithmic radiological burden categories. Importantly, because the reference categories were derived from quantitative CT characteristics rather than independently assigned by radiologists, this *κ* value should be interpreted as evidence that the model accurately reproduces the proposed imaging-based stratification rule, rather than as independent validation of clinically established VAP severity.

As shown in Figure 6b, the proposed model achieved a higher weighted κ than 3D U-Net (*κ* = 0.643), V-Net (*κ* = 0.671), nnU-Net (*κ* = 0.724), and vanilla Swin UNETR (*κ* = 0.762). These comparisons demonstrate improved consistency in predicting the predefined ordinal radiological burden categories across model architectures.

The linear regression analysis and scatter plots of Figure 6c displayed a correlation between a predicted severity score derived from the imaging grading criteria used in this study with the Clinician's APACHE II critical illness score (Spearman's *ρ* = 0.74, 95% CI: 0.69–0.79, *P* < 0.001). The three-point grading score system used to group the patients clearly demonstrated a stepwise stratification relative to the APACHE II total score, with little overlap between the three groups but a consistent overall trend of each grouping. In particular, the width of the 95% confidence band around the regression line illustrates that a statistically stable linear fit exists between the imaging grading criteria used in this study and those used by clinicians to evaluate critically ill patients.

According to data in Figure 6d, the CPIS score dimension box plot further validated its clinical discriminant validity in terms of representative function (i.e., to measure the severity of pneumonia). In the comparison of median CPIS scores among three groups (i.e., mild, moderate, and severe), the score showed an increase of 5.1 for Mild CPIS score, 7.1 for Moderate CPIS score and 9.2 for Severe CPIS score groups. The Kruskal–Wallis analysis between groups was significantly different from each other at the H = 38.62, *P* < 0.001 level. The *post-hoc* two-tailed analyses showed statistically different between Mild and Moderate CPIS score groups at a *P*-value <0.001, and between Moderate and Severe CPIS score groups at a *P*-value <0.01 level. CPIS score quartiles of the Severe group were found to be significantly lower than the quartile range (narrower interquartile ranges), indicating the overall higher clustering ability of inflammatory markers in Severe group patients with severe infection.

### 5.4 Ablation experiment results

To quantitatively analyze the independent contributions of each core module to segmentation accuracy, classification discriminant power, grading consistency, and clinical prognostic correlation, Table 3 summarizes the comprehensive performance comparison results of the eight ablation configurations on the internal validation set.

**Table 3:** Ablation study results on internal validation set.

Configuration	Dice (95% CI) ↑	Macro AUC (95% CI) ↑	Weighted κ (95% CI) ↑	28-day mortality OR (95% CI)
A: Ours (Full Model)	**0.847** (**0.839–0.855)**	**0.919** (**0.894–0.941)**	**0.826** (**0.748–0.892)**	**3.17** (**1.82–5.51)**
B: w/o Anatomical Prior (1-ch input)	0.815 (0.806–0.824)	0.897 (0.868–0.923)	0.781 (0.692–0.855)	2.68 (1.54–4.67)
C: CT-only (w/o clinical parameter fusion; sensitivity analysis)	0.831 (0.822–0.840)	0.905 (0.878–0.929)	0.789 (0.702–0.860)	2.41 (1.38–4.21)
D: w/o Focal-Tversky (Dice Loss only)	0.802 (0.792–0.812)	0.911 (0.884–0.935)	0.814 (0.731–0.882)	3.02 (1.73–5.27)
E: Single-task (Seg. only)	0.838 (0.830–0.846)	—	—	—
F: Single-task (Cls. only)	—	0.903 (0.875–0.928)	—	—
G: Single-task (Grade only)	—	—	0.793 (0.705–0.863)	2.74 (1.57–4.79)
H: Backbone → 3D ResNet-50	0.796 (0.785–0.807)	0.872 (0.839–0.901)	0.714 (0.611–0.801)	2.33 (1.34–4.06)
I: Backbone → 3D ConvNeXt	0.818 (0.808–0.828)	0.891 (0.861–0.917)	0.756 (0.661–0.834)	2.52 (1.45–4.38)
J: Training from Scratch	0.819 (0.809–0.829)	0.896 (0.867–0.921)	0.784 (0.695–0.856)	2.56 (1.47–4.46)

↑ indicates higher is better. Bold denotes the best result (Full Model, config A). “—” indicates the metric is not applicable for the given single-task configuration. OR, Odds Ratio for 28-day mortality derived from univariate logistic regression of model grading output. All experiments use the same training data, preprocessing, and hyperparameters; only the specified module is modified. Configs E–G train independent networks for each task; configs H–I replace only the encoder backbone while preserving the multi-task heads and Cross-Attention fusion.

Table 3 displays the original configuration (Model A) yielding the best results on all four evaluation criteria evaluated. All individual modules exhibited diverse effects on the evaluation metrics. Anatomical prior (A vs. B) contributed to a Dice increase of 0.032 as well as to the Kappa increase of 0.045. This indicates that the lobar mask provided an important anatomic reference for localizing lesions against the anatomic boundaries when compared to configuration B with no lobar mask being used as a reference. Ventilation parameter fusion (A vs. C) improved Dice and weighted κ, suggesting that physiological information provides complementary context for CT-based radiological burden assessment. However, these ventilation variables may reflect overall respiratory dysfunction and critical illness severity rather than VAP-specific pathophysiology. Focal Tversky loss (A vs. D) yielded a Dice increase of 0.045 as the combination of Focal's small-spot penalty mechanism and Tversky's false-negative weighting method together diminished the potential for extreme foreground/background imbalance in evaluating the performance of the module. Multi-task joint learning (A vs. E/F/G) improved the segmentation Dice from 0.838 to 0.847 and the grading Kappa from 0.793 to 0.826, demonstrating a significant positive transfer effect and regularization benefit among the three complementary tasks. Backbone network comparison (A vs. H/I) showed that the Swin Transformer improved Dice by 0.051 compared to 3D ResNet-50 and by 0.029 compared to 3D ConvNeXt, validating the Transformer architecture's long-range modeling advantage in capturing global features such as diffuse GGO.

The contribution of source-domain pretraining was further examined by training the complete architecture from random initialization (configuration J). Without MosMedData and COVID-19-CT-Seg pretraining, Dice decreased from 0.847 to 0.819, radiological-sign macro AUC from 0.919 to 0.896, and weighted κ from 0.826 to 0.784. Thus, source-domain pretraining improved Dice, macro AUC, and weighted κ by 0.028, 0.023, and 0.042, respectively. The consistent improvements across all three tasks indicate that pneumonia-related source-domain representations provide useful initialization for the relatively small VAP cohort, while the remaining performance gain still depends on VAP-specific fine-tuning and multimodal learning.

As a conservative sensitivity analysis, configuration C excluded all clinical variables and therefore relied exclusively on CT information available at the imaging time. Performance remained stable (Dice = 0.831, macro-AUC = 0.905, weighted *κ* = 0.789), indicating that the principal findings were not dependent on potentially time-sensitive clinical inputs.

### 5.5 External validation and clinical survival

To provide an initial assessment of cross-center and cross-scanner robustness, Figure 7 evaluates performance in the independent external cohort together with scanner-specific subgroup results and survival outcomes.

**Figure 7:**
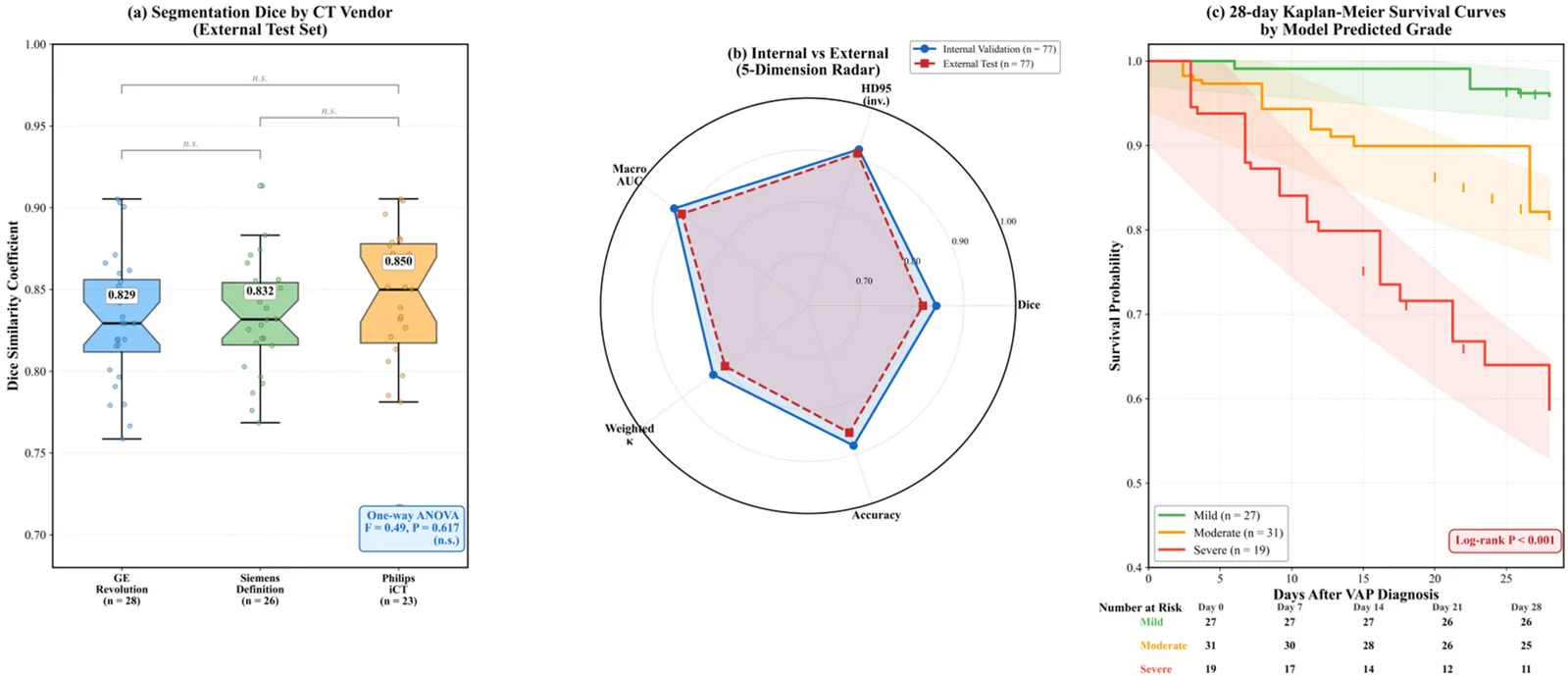
Generalization performance and clinical survival analysis. Inverse normalization was performed on HD95: $\mathrm{HD}95_{inv}=1\text{-}\frac{\mathrm{HD}95}{100}$. **(a)** Segmentation dice by CT vendor (external test set); **(b)** internal vs external (5-dimension radar); **(c)** 28-day Kaplan-Meier survival curves by model predicted grade.

As shown in Figure 7, the model maintained broadly comparable performance in the external cohort. Median Dice values were 0.829, 0.832, and 0.850 for GE, Siemens, and Philips scanners, respectively, with no statistically significant difference among the three subgroups (ANOVA F = 0.49, *P* = 0.617). Given the limited subgroup sizes, these findings should be interpreted as preliminary evidence of cross-scanner robustness rather than definitive proof of device-independent generalizability. Isotropic resampling and HU normalization in the preprocessing stage effectively suppressed differences in imaging parameters between devices. Figure 7b shows a five-dimensional radar chart that visually compares the overall performance of the internal validation set and the external test set: the five core metrics (Dice, HD95, AUC, Kappa, Accuracy) on the external test set were 0.821, 9.14 mm, 0.901, 0.798, and 85.7%, respectively, showing only a slight decrease compared to the internal validation set (0.847, 8.32 mm, 0.919, 0.826, and 88.3%). The substantial overlap of the radar profiles provides preliminary evidence that model performance was preserved across the two participating centers. Figure 7c shows the Kaplan–Meier survival curves, which are the core evidence for validating the clinical value of this study: the 28-day survival curves of the three groups of patients based on the model grading show clear stratification, with a log-rank test *P* < 0.001, proving that the severity grading output by the model is a strong independent predictor of 28-day mortality and has the potential to directly guide clinical decision-making.

### 5.6 Clinical value validation and discussion

#### 5.6.1 Incremental prognostic value of the imaging-derived radiological burden measure

Because the proposed radiological burden categories were algorithmically derived from CT characteristics, the following analyses were designed as external clinical association analyses rather than as validation against an independent clinical severity reference standard. Associations with APACHE II, CPIS, and 28-day mortality were examined to determine whether the imaging-derived burden stratification carries clinically relevant information beyond its underlying radiological definition, as shown in Table 4.

**Table 4:** Multivariable cox proportional hazards regression for 28-day mortality.

Variable	Univariate HR (95% CI)	*P* value	Multivariable HR (95% CI)	*P* value
Age (per 10-year increase)	1.38 (1.12–1.70)	0.003	1.24 (0.98–1.57)	0.072
Sex (Male vs. Female)	1.15 (0.74–1.79)	0.526	—	—
BMI (per 5 kg/m^2^ increase)	0.91 (0.72–1.15)	0.431	—	—
APACHE II (per 5-point increase)	1.82 (1.48–2.24)	<0.001	1.45 (1.13–1.86)	0.004
SOFA (per 3-point increase)	1.64 (1.31–2.05)	<0.001	1.18 (0.89–1.57)	0.248
CPIS (per 2-point increase)	1.41 (1.10–1.81)	0.007	1.12 (0.84–1.49)	0.437
Ventilation duration (per 48 h increase)	1.29 (1.08–1.54)	0.005	1.16 (0.95–1.42)	0.153
PaO₂/FiO₂ ratio (per 50 decrease)	1.47 (1.19–1.82)	<0.001	1.21 (0.94–1.56)	0.138
Model Severity Grade (ordinal: Mild/Moderate/Severe)	2.86 (1.94–4.22)	<0.001	**2.34** (**1.52–3.61)**	**<0**.**001**

Univariate analysis included all 512 cases. Multivariable model retained variables with univariate *P* < 0.10 via backward stepwise selection (entry *P* < 0.05, removal *P* > 0.10). “—” indicates the variable was excluded from the multivariable model due to univariate *P* > 0.10. The proportional hazards assumption was verified by Schoenfeld residual test (*P* = 0.384). The model concordance index (C-index) was 0.79 (95% CI: 0.74–0.84). The Model Severity Grade was entered as a continuous ordinal variable (Mild = 1, Moderate = 2, Severe = 3); HR represents the risk increase per one-grade elevation.

HR, hazard ratio from Cox proportional hazards regression; CI, confidence interval.

Bold values indicate the adjusted hazard ratio and corresponding *P* value for the model-derived severity grade, highlighting its statistically significant association with 28-day mortality after adjustment for clinical covariates.

To determine whether the imaging-derived radiological burden measure provides incremental prognostic information beyond established clinical variables, two nested Cox proportional hazards models were compared. The clinical model included age, APACHE II, SOFA, CPIS, ventilation duration, and PaO₂/FiO₂ ratio as prespecified covariates. The extended model additionally included the imaging-derived radiological burden grade. To avoid instability associated with data-driven variable selection, all prespecified covariates were retained without backward stepwise elimination. Model discrimination, calibration, likelihood-ratio improvement, and clinical net benefit were compared between the two models.

After adjustment for age, APACHE II, SOFA, CPIS, ventilation duration, and oxygenation status, a one-level increase in the model-predicted radiological burden grade remained associated with increased 28-day mortality (HR = 2.34, 95% CI: 1.52–3.61, *P* < 0.001). This finding provides evidence of prognostic association and supports the potential clinical relevance of the imaging-derived burden score; however, it does not establish the proposed categories as independently validated clinical VAP severity grades.

As shown in Table 5, addition of the imaging-derived radiological burden grade improved prognostic performance beyond the prespecified clinical model. The C-index increased from 0.744 (95% CI: 0.695–0.793) to 0.790 (95% CI: 0.740–0.840), while the 28-day time-dependent AUC increased from 0.758 to 0.816. Overall prediction error decreased, as reflected by the reduction in Brier score from 0.151 to 0.134, and the calibration slope improved from 0.94 to 0.98. The likelihood-ratio test demonstrated a significant improvement in model fit after addition of the imaging-burden measure (*χ*^2^ = 15.2, *P* < 0.001). Decision-curve analysis also indicated greater clinical net benefit for the extended model, with net benefit increasing from 0.071 to 0.094 at a 20% risk threshold. These findings suggest that quantitative CT-derived disease burden provides incremental prognostic information beyond established clinical severity measures, rather than demonstrating that the AI model itself independently determines mortality risk.

**Table 5:** Incremental prognostic performance of the imaging-derived radiological burden measure for 28-day mortality.

Model	C-index (95% CI)	28-day time-dependent AUC (95% CI)	Calibration slope (95% CI)	Brier score	Likelihood-ratio *χ*^2^ (*P* value)	Net benefit at 20% threshold
Clinical model	0.744 (0.695–0.793)	0.758 (0.708–0.808)	0.94 (0.84–1.04)	0.151	Reference	0.071
Clinical + imaging-burden model	0.790 (0.740–0.840)	0.816 (0.771–0.861)	0.98 (0.89–1.07)	0.134	15.2 (<0.001)	0.094

The clinical model included age, APACHE II, SOFA, CPIS, ventilation duration, and PaO₂/FiO₂ ratio. The extended model additionally included the imaging-derived radiological burden grade as an ordinal variable. C-index and time-dependent AUC quantify discrimination; a calibration slope approaching 1.0 indicates better agreement between predicted and observed risk; lower Brier scores indicate better overall prediction accuracy. The likelihood-ratio test compares the nested clinical and extended models. Decision-curve net benefit is reported at a representative 20% predicted-risk threshold. All prespecified clinical covariates were retained in both models without backward stepwise selection.

Clinical association and external validation of the proposed radiological burden stratification, as shown in Table 6.

**Table 6:** Clinical association and external validation of the proposed radiological burden stratification.

Validation dimension	Result	Statistical evidence
Association with APACHE II	Spearman *ρ* = **0.74**	95% CI: **0.69–0.79**, *P* < 0.001
CPIS – Mild burden	**5.1**	—
CPIS – Moderate burden	**7.1**	—
CPIS – Severe burden	**9.2**	—
CPIS difference across grades	H = **38.62**	*P* < 0.001
Mild vs. Moderate CPIS	—	*P* < 0.001
Moderate vs. Severe CPIS	—	*P* < 0.01
28-day mortality, univariable grade effect	HR = **2.86**	95% CI: **1.94–4.22**, *P* < 0.001
28-day mortality, adjusted grade effect	HR = **2.34**	95% CI: **1.52–3.61**, *P* < 0.001
Multivariable prognostic model	C-index = **0.79**	95% CI: **0.74–0.84**
Internal grading performance	Accuracy = **88.3%**, *κ* = **0.826**	—
External grading performance	Accuracy = **85.7%**, *κ* = **0.798**	—

The proposed radiological burden categories were defined using $V_{ratio}$, lobar involvement, and cavitation. Associations with APACHE II and CPIS were used to assess convergent clinical validity. Cox proportional hazards regression evaluated the association with 28-day mortality. The adjusted Cox model included age, APACHE II, SOFA, CPIS, ventilation duration, and PaO₂/FiO₂ ratio. External grading performance was evaluated in the independent 77-case cohort.

Bold values highlight the key quantitative findings regarding clinical associations, radiological burden stratification, mortality risk, prognostic performance, and internal and external validation. Bold formatting is used for emphasis and does not necessarily indicate statistical significance.

As summarized in Table 6, the proposed radiological burden stratification showed consistent associations with established clinical severity measures and patient outcome. The ordinal burden grade correlated strongly with APACHE II (Spearman's *ρ* = 0.74, 95% CI: 0.69–0.79, *P* < 0.001). CPIS also increased progressively across mild, moderate, and severe burden groups (5.1, 7.1, and 9.2, respectively; Kruskal–Wallis H = 38.62, *P* < 0.001). In survival analysis, each one-level increase in radiological burden was associated with a higher 28-day mortality risk in both univariable analysis (HR = 2.86, 95% CI: 1.94–4.22, *P* < 0.001) and multivariable analysis (HR = 2.34, 95% CI: 1.52–3.61, *P* < 0.001). Grading performance was also preserved in the independent external cohort (weighted *κ* = 0.798; accuracy = 85.7%), compared with *κ* = 0.826 and 88.3% accuracy internally. These findings support the clinical relevance and cross-center robustness of the proposed imaging-burden stratification, while not establishing the 15% and 35% values as definitive VAP-specific clinical cutoffs.

#### 5.6.2 Limitations

This study has several limitations. First, the retrospective cohort was derived from only two tertiary hospitals, and the external cohort was relatively small (*n* = 77), limiting broad generalizability. Second, the cohort included only clinically diagnosed VAP cases and no non-VAP ICU controls; therefore, the reported AUC reflects radiological-finding classification within VAP cases rather than diagnostic discrimination between VAP and alternative pulmonary conditions. Third, radiologists were aware that the included patients had VAP, which may introduce diagnostic expectation bias. Fourth, the three-level radiological burden categories were algorithmically derived from lesion extent and cavitation rather than independently assigned clinical severity labels. Prospective multicenter studies with non-VAP controls and independent clinical severity assessment are needed.

In addition, ventilation parameters such as PEEP, PaO₂/FiO₂, FiO₂, tidal volume, and ventilation duration are not specific to VAP and may primarily reflect overall respiratory dysfunction or critical illness severity. Their contribution should therefore be interpreted as complementary clinical context rather than VAP-specific information.

## 6 Conclusions

This study develops a multimodal deep-learning framework for quantitative CT analysis in clinically diagnosed VAP, integrating lesion segmentation, radiological-finding classification, and radiological burden grading. The model achieved a Dice score of 0.847, a macro-average AUC of 0.919, and a weighted κ of 0.826, with generally preserved performance on external validation. The radiological burden grade was also significantly associated with 28-day mortality. However, because no non-VAP controls were included, the current results should not be interpreted as evidence of VAP diagnostic discrimination. Future prospective multicenter studies are required to further establish diagnostic and clinical utility.

## Data Availability

The original contributions presented in the study are included in the article/Supplementary Material, further inquiries can be directed to the corresponding authors.

## References

[1] Howroyd F Chacko C MacDuff A Gautam N Pouchet B Tunnicliffe B. Ventilator-associated pneumonia: pathobiological heterogeneity and diagnostic challenges. Nat Commun. (2024) 15(1):6447. 10.1038/s41467-024-50805-z 39085269 PMC11291905

[2] Miron M Blaj M Ristescu AI losep G Avadanei AN Iosep D. Hospital-acquired pneumonia and ventilator-associated pneumonia: a literature review. Microorganisms. (2024) 12(1):213. 10.3390/microorganisms12010213 38276198 PMC10820465

[3] Hraiech S Ladjal K Guervilly C Hyvernat H Papazian L Forel JM. Lung abscess following ventilator-associated pneumonia during COVID-19: a retrospective multicenter cohort study. Critical Care. (2023) 27(1):385. 10.1186/s13054-023-04660-x 37794402 PMC10552315

[4] Abdelmaboud MA El Badawy MA Alkenawy MA Abdelaal AA. Comparative study of lung ultrasound versus chest X-ray in diagnosis of ventilator associated pneumonia in intensive care unit. Al-Azhar Int Med J. (2025) 2025(3):277–83. 10.21608/aimj.2025.446501

[5] Ronneberger O Fischer P Brox T. “U-net: convolutional networks for biomedical image segmentation”. In: Navab N Hornegger J Wells WM Frangi AF, editors. Medical Image Computing and Computer-Assisted Intervention—MICCAI 2015. Lecture Notes in Computer Science. Cham: Springer (2015 ). p. 234–41. 10.1007/978-3-319-24574-4_28

[6] Isensee F Jaeger PF Kohl SAA Petersen J Maier-Hein KH. nnU-Net: a self-configuring method for deep learning-based biomedical image segmentation. Nat Methods. (2021) 18(2):203–11. 10.1038/s41592-020-01008-z 33288961

[7] Hatamizadeh A Tang Y Nath V Yang D Myronenko A Landman B. UNETR: transformers for 3D medical image segmentation. In: Proceedings of the IEEE/CVF Winter Conference on Applications of Computer Vision (WACV) (2022), 574–584 10.1109/WACV51458.2022.00181

[8] Wang H Guo S Ye J Deng Z Cheng J Li T. “SAM-Med3D: towards general-purpose segmentation models for volumetric medical images”. In: Bue AD Canton C Pont-Tuset J Tommasi T, editors. Computer Vision—eCCV 2024 Workshops. Lecture Notes in Computer Science. Cham: Springer (2025). p. 49–67. 10.1007/978-3-031-91721-9_4

[9] Morozov SP Andreychenko AE Blokhin IA Gelezhe PB Gonchar AP Nikolaev AE. MosMedData: data set of 1110 chest CT scans performed during the COVID-19 epidemic. Digit Diagn. (2020) 1(1):49–59. 10.17816/DD46826

[10] Wang L Lin ZQ Wong A. COVID-net: a tailored deep convolutional neural network design for detection of COVID-19 cases from chest X-ray images. Sci Rep. (2020) 10:19549. 10.1038/s41598-020-76550-z 33177550 PMC7658227

[11] Shan F Gao Y Wang J Shi W Shi N Han M. Abnormal lung quantification in chest CT images of COVID-19 patients with deep learning and its application to severity prediction. Med Phys. (2021) 48(4):1633–45. 10.1002/mp.14609 33225476 PMC7753662

[12] Pour ZM Shirazi AAB. Identifying COVID-19-infected segments in lung CT scan through two innovative artificial intelligence-based transformer models. Arch Acad Emerg Med. (2024) 13(1):21. 10.22037/aaemj.v13i1.2515 PMC11829223 39958958

[13] Lizzi F Postuma I Brero F Cabini RF Fantacci ME Lascialfari A. Quantification of pulmonary involvement in COVID-19 pneumonia: an upgrade of the LungQuant software for lung CT segmentation. Eur Phys J Plus. (2023) 138(4):1–10. 10.1140/epjp/s13360-023-03896-4 PMC10088731 37064789

[14] Ma J Wang Y An X Ge C Yu Z Chen J. Toward data-efficient learning: a benchmark for COVID-19 CT lung and infection segmentation. Med Phys. (2021) 48(3):1197–210. 10.1002/mp.14676 33354790

[15] Blonz G Kouatchet A Chudeau N Pontis E Lorber J Lemeur A. Epidemiology and microbiology of ventilator-associated pneumonia in COVID-19 patients: a multicenter retrospective study in 188 patients in an un-inundated French region. Crit Care. (2021) 25(1):72. 10.1186/s13054-021-03493-w 33602296 PMC7891465

[16] Maes M Higginson E Pereira-Dias J Curran MD Parmar S Khokhar F. Ventilator-associated pneumonia in critically ill patients with COVID-19. Crit Care. (2021) 25(1):25. 10.1186/s13054-021-03460-5 33430915 PMC7797892

[17] Gao Y Li C Chang W Du B Ye X Yeo YH. Multi-modal AI for opportunistic screening, staging and progression risk stratification of steatotic liver disease. Nat Commun. (2026) 17:1562. 10.1038/s41467-026-68414-3 41672973 PMC12894725

[18] Huang W Pan Z Wang X Zhang Y He J Gee JC. Text-image co-alignment for weakly supervised polyp segmentation. IEEE Trans Med Imaging. (2026) 45(6):3394–408. 10.1109/TMI.2026.3674592 41843525

[19] Hong S Yang B Chen Y Quan H Liu S Tang M. Adaptive fusion neural networks for sparse-angle X-ray 3D reconstruction. Comput Model Eng Sci. (2025) 144(1):1091–112. 10.32604/cmes.2025.066165

[20] Li X Chen W Duan X Gu X Li C. Collaborative surgical instrument segmentation for monocular depth estimation in minimally invasive surgery. Med Image Anal. (2025) 106:103765. 10.1016/j.media.2025.103765 40848507

[21] Yu X Luan S Lei S Huang J Liu Z Xue X. Deep learning for fast denoising filtering in ultrasound localization microscopy. Phys Med Biol. (2023) 68(20):205002. 10.1088/1361-6560/acf98f 37703894

[22] Luan S Yu X Lei S Ma C Wang X Xue X. Deep learning for fast super-resolution ultrasound microvessel imaging. Phys Med Biol. (2023) 68(24):245023. 10.1088/1361-6560/ad0a5a 37934040

[23] Yuan H Wang W Ma J Li H Reis RL Chen Y. Inhalable PD-L1-engineered hybrid cellular vesicles suppress excessive neutrophil activation and restore mitochondrial homeostasis to alleviate ischemia-reperfusion lung injury and pneumonia. Bioact Mater. (2026) 62:319–41. 10.1016/j.bioactmat.2026.03.024 41884518 PMC13011060

[24] Liang Y Zhu C Tian C Lin Q Li Z Li Z. Early prediction of ventilator-associated pneumonia in critical care patients: a machine learning model. BMC Pulm Med. (2022) 22(1):250. 10.1186/s12890-022-02031-w 35752818 PMC9233772

[25] Jain G Mishra SB Agarwal A Talawar P Panda S. Lung ultrasound score as a predictor of clinical severity and prognosis in patients of ventilator-associated pneumonia. Indian J Crit Care Med. (2024) 28(11):1009. 10.5005/jp-journals-10071-24828 39882053 PMC11773579

[26] Panda S Agarwal A Mishra SB Jain G Talawar P. Opportunistic use of chest low-dose computed tomography (LDCT) imaging for low bone mineral density and osteoporosis screening: cutoff thresholds for the attenuation values of the lower thoracic and upper lumbar vertebrae. Quant Imaging Med Surg. (2024) 14(7):4792–803. 10.21037/qims-24-59 39022254 PMC11250341

[27] Choi H Kim H Jin KN Jeong YJ Chae KJ Lee KH. A challenge for emphysema quantification using a deep learning algorithm with low-dose chest computed tomography. J Thorac Imaging. (2022) 37(4):253–61. 10.1097/RTI.0000000000000647 35749623

[28] Thomas MA Laforest R Karageorgiou J Giardina D Fraum TJ Malone CD. Addressing lung truncation in 99mTc-MAA SPECT/CT for 90Y microsphere radioembolization treatment planning. EJNMMI Phys. (2024) 11(1):104. 10.1186/s40658-024-00705-4 39692992 PMC11655778

[29] Xie K Gao L Zhang H Zhang S Xi Q Zhang F. Inpainting truncated areas of CT images based on generative adversarial networks with gated convolution for radiotherapy. Med Biol Eng Comput. (2023) 61(7):1757–72. 10.1007/s11517-023-02809-y 36897469

[30] Wasserthal J Breit HC Meyer MT Pradella M Hinck D Sauter AW. TotalSegmentator: robust segmentation of 104 anatomic structures in CT images. Radiol Artif Intell. (2023) 5(5):e230024. 10.1148/ryai.230024 37795137 PMC10546353

[31] Wu G Fu Y Feng W Liu C Li J Gao H. Prevalence and risk factors for ventilator-associated pneumonia after cardiac surgery: a systematic review and meta-analysis. J Thorac Dis. (2024) 16(9):5946–57. 10.21037/jtd-24-324 39444916 PMC11494587

[32] Tusor N De Cunto A Basma Y Klein JL Meau-Petit V. Ventilator-associated pneumonia in neonates: the role of point of care lung ultrasound. Eur J Pediatr. (2021) 180(1):137–46. 10.1007/s00431-020-03710-8 32592026 PMC7317892

[33] Kheirdoust A Barzanouni F Rasoulian A Behrouzi F Esmailzadeh A Ghaddaripouri K. Evaluation of machine learning methods developed for prediction and diagnosis of pneumonia: a systematic review. Health Sci Rep. (2025) 8(12):71446. 10.1002/hsr2.71446 PMC12665151 41328175

[34] Ganesan SK Velusamy P Rajendran S Sakthivel R Bose M Inbaraj BS. ZooCNN: a zero-order optimized convolutional neural network for pneumonia classification using chest radiographs. J Imaging. (2025) 11(1):22. 10.3390/jimaging11010022 39852335 PMC11765744

[35] He Y Yun J Li J Wang Y Xu J. Multicenter deep learning study for lung tumor subtype segmentation in CT images using Swin UNETR, nnU-Net, and TransUNet. J Radiat Res Appl Sci. (2025) 18(3):101721. 10.1016/j.jrras.2025.101721

[36] Tang Z Zhang J Bai C Zhang Y Liang K Yao X. Dense Swin-UNet: dense Swin transformers for semantic segmentation of pneumothorax in CT images. J Mech Med Biol. (2023) 23(08):2340069. 10.1142/S0219519423400699

[37] Hansell DM Bankier AA MacMahon H McLoud TC Müller NL Remy J. Fleischner Society: glossary of terms for thoracic imaging. Radiology. (2008) 246(3):697–722. 10.1148/radiol.2462070712 18195376

[38] Borghesi A Maroldi R. COVID-19 outbreak in Italy: experimental chest X-ray scoring system for quantifying and monitoring disease progression. Radiol Med. (2020) 125(5):509–13. 10.1007/s11547-020-01200-3 32358689 PMC7194501

[39] Yang R Li X Liu H Zhen Y Zhang X Xiong Q. Chest CT severity score: an imaging tool for assessing severe COVID-19. Radiol Cardiothorac Imaging. (2020) 2(2):e200047. 10.1148/ryct.2020200047 33778560 PMC7233443

[40] Yeap PL Wong YM Ong ALK Tuan JKL Pang EPP Park SY. Predicting dice similarity coefficient of deformably registered contours using Siamese neural network. Phys Med Biol. (2023) 68(15):155016. 10.1088/1361-6560/ace6f0 37437590

[41] Tanaka R Matsumoto I Tamura M Takata M Yoshida S Saito D. Dynamic chest radiography: clinical validation of ventilation and perfusion metrics derived from changes in radiographic lung density compared to nuclear medicine imaging. Quant Imaging Med Surg. (2021) 11(9):4016. 10.21037/qims-20-1217 34476186 PMC8339647

[42] Gholamiankhah F Mostafapour S Goushbolagh NA Shojaerazavi S Layegh P Tabatabaei SM. Automated lung segmentation from computed tomography images of normal and COVID-19 pneumonia patients. Iran J Med Sci. (2022) 47(5):440. 10.48550/arXiv.2104.02042 36117575 PMC9445870

[43] Khalal DM Behouch A Azizi H Maalej N. Automatic segmentation of thoracic CT images using three deep learning models. Cancer Radiother. (2022) 26(8):1008–15. 10.1016/j.canrad.2022.02.001 35803861

[44] Khomduean P Phuaudomcharoen P Boonchu T Taetragool U Chamchoy K Wimolsiri N. Segmentation of lung lobes and lesions in chest CT for the classification of COVID-19 severity. Sci Rep. (2023) 13(1):20899. 10.1038/s41598-023-47743-z 38017029 PMC10684885

[45] Zhou T Canu S Ruan S. Automatic COVID-19 CT segmentation using U-Net integrated spatial and channel attention mechanism. Int J Imaging Syst Technol. (2021) 31(1):16–27. 10.1002/ima.22527 33362345 PMC7753491

[46] Rodriguez-Leal CM González-Corralejo C Candel FJ Salavert M. Towards a holistic approach to pulmonary infections. Insights from the sixth annual meeting of Spanish experts 2024. Rev Esp Quimioter. (2025) 38(3):152. 10.37201/req/017.2025 40145524 PMC12095940

[47] Guleria R Chawla R Modi M Rodrigues C Jaiswal A Saxena P. ISCCM position statement on the approach to and management of critically ill patients with tuberculosis. Indian J Crit Care Med. (2024) 28(Suppl 2):67. 10.5005/jp-journals-10071-24783 PMC11369919 39234233

